# Chlorhexidine vs Routine Foot Washing to Prevent Diabetic Foot Ulcers: A Randomized Clinical Trial

**DOI:** 10.1001/jamanetworkopen.2024.60087

**Published:** 2025-02-18

**Authors:** Alison D. Lydecker, Justin J. Kim, Gwen L. Robinson, J. Kristie Johnson, Clayton H. Brown, Christopher C. Petruccelli, Michael L. Terrin, David J. Margolis, Mary-Claire Roghmann

**Affiliations:** 1 Baltimore Veterans Affairs (VA) Medical Center, VA Maryland Health Care System, Baltimore; 2 Department of Epidemiology and Public Health, University of Maryland School of Medicine, Baltimore; 3 Department of Pathology, University of Maryland School of Medicine, Baltimore; 4 Department of Internal Medicine, University of Maryland School of Medicine, Baltimore; 5 Department of Dermatology, Perelman School of Medicine, University of Pennsylvania, Philadelphia

## Abstract

**Importance:**

Foot ulcers are a common and feared complication for people with diabetes because 20% of foot ulcers become infected and lead to a lower extremity amputation.

**Objective:**

To evaluate the effect of daily foot care using chlorhexidine wipes vs soap-and-water wipes for 1 year on the risk of developing new foot complications in veterans with diabetes.

**Design, Setting, and Participants:**

This double-blind, placebo-controlled, phase 2b randomized clinical trial was conducted at the Baltimore Veterans Affairs (VA) Medical Center between January 2019 to January 2023. Veterans were eligible if they had a diabetes diagnosis, were at high risk for diabetic foot complications, were ambulatory, had both feet, and did not have a current foot infection. Participants were randomly assigned 1:1 to receive either soap-and-water wipes (control group) or 2% chlorhexidine wipes (chlorhexidine group). Intention-to-treat data analysis was conducted from October 5, 2023, to April 24, 2024.

**Intervention:**

Daily use of a 2% chlorhexidine wipe or a soap-and-water wipe on the feet for 1 year. Wipes were nearly identical in color, size, shape, thickness, feel, and scent. Both chlorhexidine and control groups received the same lotion for application on the feet after wipe use and education on foot self-care.

**Main Outcomes and Measures:**

The primary outcome was time in days from randomization to new foot complication, including chronic foot ulcer, foot infection, or foot amputation.

**Results:**

A total of 175 participants (170 males [97%]; mean [SD] age at enrollment, 68 [9] years; 1 Asian [1%], 117 Black or African American [67%], 53 White [30%] individuals) were randomly assigned to the chlorhexidine group (n = 88) or the control group (n = 87). Twelve participants (14%) in the chlorhexidine group and 14 participants (16%) in the control group developed a new foot complication within 1 year. Median (IQR) time from randomization to development of a new foot complication was 232 (115-315) days. The reduction in hazard of new foot complications in the chlorhexidine group compared with the control group was not significant (hazard ratio, 0.83; 95% CI, 0.39-1.80). The intervention was well tolerated, with 145 participants (83%) continuing it over the study period. Sixty adverse events occurred, but none was related to the study products or procedures.

**Conclusions and Relevance:**

This randomized clinical trial found that daily use of chlorhexidine wipes for foot washing for 1 year did not lead to a significant reduction in the risk of new foot complications compared with daily use of soap-and-water wipes. The intervention was well tolerated, and the trial provides important lessons for future studies on diabetic foot ulcer prevention.

**Trial Registration:**

ClinicalTrials.gov Identifier: NCT03503370

## Key Points

**Question:**

What is the effect of daily use of chlorhexidine wipes vs soap-and-water wipes on the feet for 1 year on the risk of developing a new foot complication?

**Findings:**

In this randomized clinical trial involving 175 veterans with diabetes, use of chlorhexidine wipes did not significantly decrease the risk of new foot complications compared with soap-and-water wipes. Veterans with a history of foot complications had the highest risk of new foot complications.

**Meaning:**

Although there was no difference in outcomes between the 2 groups, the findings indicate that the intervention was well tolerated, and the trial provides important lessons for future studies on diabetic foot ulcer prevention.

## Teaser

This randomized clinical trial examines the effectiveness of 2 foot care interventions for veterans with diabetes at risk for developing chronic foot ulcer, foot infection, or other complications leading to foot amputation.

## Introduction

Foot ulcers are a common and feared complication for people with diabetes. In the US, more than 1 in 10 adults have diabetes.^1^ Each year 1 in 20 people with diabetes develop a foot ulcer,^2^ with 20% of these individuals going on to have a lower extremity amputation, which can decrease their mobility and independence.

People who recover from a diabetic foot ulcer are considered in remission. They are at high risk for ulcer recurrence because the comorbidities underlying their ulcer have not changed.^3^ The pathway to ulceration is well established.^4^ A foot ulcer typically begins with minor trauma to the foot in a patient with diabetic peripheral neuropathy. The minor skin trauma fails to heal due to the complications of diabetes, resulting in a skin ulcer. The ulcer leads to infection, which often results in an amputation.

The role of the skin microbiota in the development of foot ulcers is unknown. Multiple skin disorders and infections are hypothesized to result from imbalance in the microbiota and the host inflammatory and immune responses.^5,6,7^ Impaired wound healing is hypothesized to be, in part, associated with dysregulated inflammation and microbial colonization,^8,9^ suggesting a possible mechanism by which the skin microbiota could promote faulty wound healing after a minor trauma leading to a foot ulcer. Prior work has shown that the feet of veterans with diabetes have an increased *Staphylococcus aureus* load compared with the feet of veterans without diabetes.^10^ If so, altering the skin microbiota of the feet could reduce the risk of foot ulcer recurrence.

Topical chlorhexidine can reduce the bacterial and fungal load, including skin pathogens such as *S aureus*. It is a broad-spectrum, commonly used antiseptic agent with an excellent safety profile. It prevents health care–associated infections, such as central line–associated bacteremia, after iatrogenic skin breakdown through reducing the microbial load on the skin.^11,12,13^ Thus, we conducted a clinical trial with the objective of evaluating the effect of daily foot care using chlorhexidine wipes vs soap-and-water wipes for 1 year on the risk of developing new foot complications (including chronic foot ulcer, foot infection, or foot amputation) in veterans with diabetes. We also aimed to determine if the chlorhexidine intervention increased chlorhexidine resistance in diabetic foot infection pathogens.

## Methods

### Trial Design and Randomization

We conducted the Preventing Diabetic Foot Ulcers Through Cleaner Feet trial as a single-center, double-blind (participant, outcome assessor), placebo-controlled, parallel group, phase 2b randomized clinical trial (RCT) at the Baltimore Veterans Affairs (VA) Medical Center. Enrollment occurred between January 29, 2019, and December 7, 2021, with 1 year of follow-up. This RCT was approved by the institutional review board of the University of Maryland Baltimore and the VA Research and Development Committee of the VA Maryland Health Care System (VAMHCS). Recruitment involved obtaining written informed consent from participating veterans enrolled in the VAMHCS. We vouch for the accuracy and completeness of the data and for the fidelity of the trial to the protocol (Supplement 1). We followed the Consolidated Standards of Reporting Trials (CONSORT) reporting guideline.

**Figure 1.**
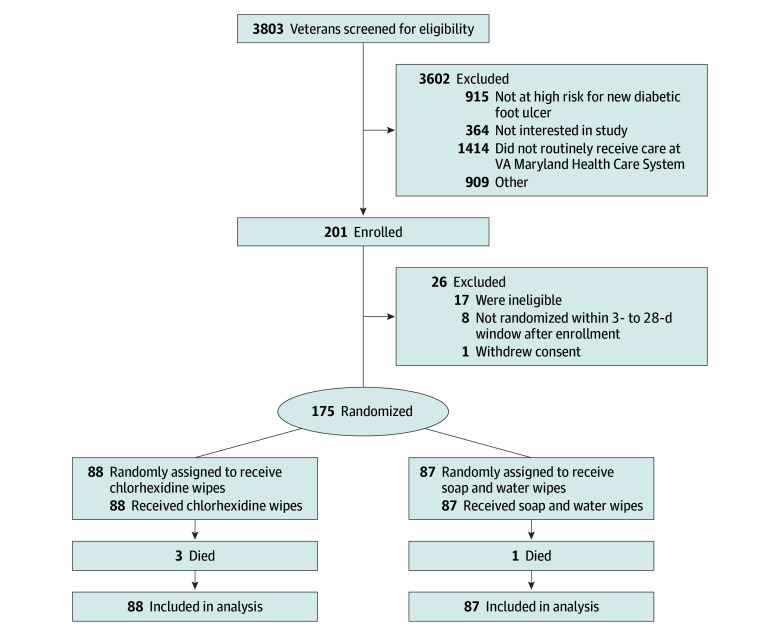
Trial Flow Diagram

Enrolled participants who met eligibility criteria and returned for a randomization visit were randomly assigned, in a 1:1 ratio, to receive either wipes containing soap and water (control group) or wipes containing 2% chlorhexidine (chlorhexidine group) (Figure 1). Randomization was not stratified. The VAMHCS Geriatric Research Education and Clinical Center Biostatistics group generated the randomization sequence using a computer-generated permuted block scheme with random block sizes and a maximum block size of 6. Study physicians, clinical coordinators, microbiology laboratory technicians, the principal investigator, and participants were blinded to the treatment assignment.

### Intervention

In the control group, participants received comfort bath wipes (Sage; Stryker) soaked in mild, rinse-free cleansers and moisturizers for daily use on their feet. In the chlorhexidine group, participants received 2% chlorhexidine gluconate wipes (Sage; Stryker), a US Food and Drug Administration–approved formulation and application of the antiseptic topical 2% chlorhexidine, for daily use on their feet. A single wipe of chlorhexidine or soap and water was applied to the feet once daily at a time chosen by the participant.

Both wipe types are available without a prescription and are nearly identical in color, size, shape, thickness, feel, and scent. Study wipes were repackaged, blinded, and tested as described in the eMethods in Supplement 2. Both groups received the same lotion for application on the feet after wipe use and education on foot self-care. All products were applied to the feet by the participant or a caregiver daily for up to 1 year (eMethods in Supplement 2). All products were purchased with grant funds and were provided free of charge to the participants.

### Recruitment and Eligibility Criteria

Eligibility requirements included age of 18 years or older; clinical diagnosis of diabetes; and at risk for a new diabetic foot ulcer due to at least 1 of the following: (1) history of diabetic foot ulcer; (2) history of major foot surgery; (3) history of moderate to severe foot infection; (4) peripheral neuropathy, onychomycosis, and hemoglobin A_1C_ higher than 8% (to convert to the proportion of total hemoglobin, multiply by 0.01); (5) peripheral neuropathy and peripheral artery disease (PAD); (6) dialysis; (7) Charcot foot; or (8) PAD requiring surgery or stenting. Within the first year of the study, the eligibility criteria were expanded to include those at high risk for a new diabetic foot ulcer^14^ but without a previous foot complication (items 4, 5, 6, 7, and 8 on the eligibility list) due to slower than expected recruitment. Key exclusion criteria were current foot infection, planned foot surgery, being nonambulatory, nursing home care, and allergy to chlorhexidine at recruitment.

### Follow-Up

Participants were followed up for a new foot complication for up to 1 year after randomization. In-person visits in a VAMHCS research clinic occurred at recruitment; randomization; and at months 3, 6, 9, 12, and 13 after randomization. These visits included data collection from the participant, swab collection from the participant’s feet, and taking photographs of the participant’s feet. The final visit at month 13 included an exit interview. If a participant developed a new foot complication before the end of the study period, this final visit occurred approximately 4 weeks after the participant stopped using the intervention. Participants received $50 compensation for each completed visit. The final study visit occurred on January 6, 2023.

All participants were contacted weekly with a reminder to use study wipes and lotion daily. All participants were contacted monthly and asked to report allergic reactions to the study wipes and hospitalizations or clinic visits for foot complications. Medical records and foot photographs for each participant were reviewed every 3 months for a new foot complication.

### Data and Swab Collection

Data on demographics, eligibility criteria, health conditions, and current and past foot characteristics were collected at baseline. Race and ethnicity were ascertained from the participant’s medical record and were collected in this analysis to characterize the study population for purposes of external generalizability. Information on usual foot care and current foot health was taken at all study visits. At each visit after starting the intervention, participants reported adherence to the intervention, and staff assessed the remaining product.

Cultures were performed with a nylon flocked swab (ESwab; Copan Diagnostics Inc) along the length of the plantar aspect of both feet at the visit 13 months after randomization (approximately 4 weeks after stopping the intervention). Swabs were worked up for *Enterobacter* spp, *S aureus*, *Klebsiella pneumoniae, Acinetobacter baumannii, Pseudomonas aeruginosa,* and *Enterococcus faecium* (ESKAPE) pathogens as well as other diabetic foot infection (DFI) pathogens (*Escherichia coli, Enterococcus faecalis*, and *Streptococcus* spp). Additionally, participants had a culture collected from the plantar aspect of both feet during each in-person study visit to test for the presence of chlorhexidine. The eMethods in Supplement 2 provide details on swab workup.

### Trial Outcomes

The primary outcome was time in days from randomization until a new chronic (present 28 days from initial diagnosis^15^) foot ulcer or wound, a moderate or severe foot infection (as defined in the Infectious Diseases Society of America Diabetic Foot Infection Severity Classification^16^) not from an existing ulcer, or a foot amputation from a new ulcer. The presence of a new foot complication was assessed from medical records, and the sequelae for those with the outcome were determined. New foot complications were confirmed by 2 physicians with expertise in infectious diseases (including M-C.R., J.J.K., and C.C.P.). All outcomes were assessed based on the first event per participant. Participants were followed up for the primary outcome until one of the following occurred: death, self-withdrawal from the study and specific request for data collection to stop, development of the primary outcome, or reaching 1 year after randomization.

The secondary outcome was susceptibility to chlorhexidine among bacterial pathogens swabbed from the feet approximately 4 weeks after stopping the intervention. Chlorhexidine minimum inhibitory concentration (MIC) for each pathogen was normalized by subtracting the median chlorhexidine MIC (MIC 50) in the literature for each pathogen (eTable in Supplement 2) from the observed MIC on a log_2_ scale.

### Statistical Analysis

We hypothesized that the chlorhexidine group would have a longer time to new foot complication than the control group. Kaplan-Meier curves were used to describe the distribution of time to new foot complication for the 2 treatment arms. The log-rank test was used to test the null hypothesis of no difference in the distribution of time to new foot complication between the 2 arms. Death was treated as a noninformative censoring mechanism. Effect size was measured with the hazard ratio (HR) and 95% CI from a simple, unadjusted Cox proportional hazards regression model. A participant’s medical record was monitored for new foot complications even if the patient stopped using the study wipes. Hence, the primary analysis followed the intention-to-treat (ITT) principle.

We hypothesized that the chlorhexidine group would not be colonized with ESKAPE and other DFI pathogens with a higher chlorhexidine MIC than the control group. All participants colonized with ESKAPE and other DFI pathogens at the final study visit were included. MICs were normalized by subtracting the literature-derived MIC 50 from the observed MIC on a log_2_ scale; for example, a normalized MIC of 1 indicated a 2-fold greater MIC compared with the literature value, or a normalized value of −1 indicated a 2-fold lesser MIC compared with the literature value. The null hypothesis of no difference in MIC distribution was tested with the Wilcoxon rank sum test due to the range-limited, discrete distributions of MICs. Effect sizes were expressed in means on a log_2_ scale with 95% CIs.

Sample size was based on the primary outcome. We tested the null hypothesis for the primary outcome that there was no difference in time to new foot complication between treatment groups using a log-rank test (2-tailed, 5% significance level). We assumed that 10% of participants would die prior to 1 year of follow-up. We used the Freedman method for sample size calculation reported in Machin et al^17^ using the PASS software (NCSS). Prior data suggested that the annual proportion of individuals with new foot complications among controls would be approximately 0.4. We assumed the true proportion for experimental participants would be 0.2 one year after randomization. With power of 0.82, the required sample size in each treatment arm to detect this difference based on the log-rank test was 100 (ie, total N = 200). Full details of the trial design and analytic approach are provided in Supplement 1.

All statistical tests were 2-tailed, and *P* < .05 was considered statistically significant. All statistical analyses were conducted using Stata 15 (StataCorp LLC), SAS 9.4 (SAS Institute Inc), and R 4.2.1 (R Project for Statistical Computing). ITT data analysis was conducted from October 5, 2023, to April 24, 2024.

## Results

The baseline demographics and health characteristics of the 175 participants were similar between treatment arms (chlorhexidine: n = 88; control: n = 87) (Table 1). Figure 1 shows the randomization and follow-up of all participants. All participants who were randomly assigned to a treatment received that treatment. Participants included 170 males (97%) and 5 females (3%); had a mean (SD) age at enrollment of 68 (9) years; and included 1 American Indian or Alaska Native (1%), 1 Asian (1%), 117 Black or African American (67%), 1 Native Hawaiian or Other Pacific Islander (1%), and 53 White (30%) individuals, of whom 174 identified as non-Hispanic or Latino (99%).

**Table 1.** Baseline Demographic and Health Characteristics of Participants by Treatment Group

Characteristic	Participants, No. (%)		Total (N = 175)
	Chlorhexidine group (n = 88)	Control group (n = 87)	
Demographics			
Age at enrollment, mean (SD), y	67 (8)	68 (10)	68 (9)
Sex			
Female	3 (3)	2 (2)	5 (3)
Male	85 (97)	85 (98)	170 (97)
Race^a^			
American Indian or Alaska Native	0	1 (1)	1 (1)
Asian	0	1 (1)	1 (1)
Black or African American	60 (68)	57 (66)	117 (67)
Native Hawaiian or Other Pacific Islander	1 (1)	0	1 (1)
White	25 (28)	28 (32)	53 (30)
Prefer not to answer	2 (2)	0	2 (1)
Ethnicity^a^			
Hispanic or Latino	0	0	0
Non-Hispanic or Latino	87 (99)	87 (100)	174 (99)
Prefer not to answer	1 (1)	0	1 (1)
Lives alone	25 (28)	28 (32)	53 (30)
Diabetes-related factors			
Most recent BMI, mean (SD)	32 (6)	31 (6)	32 (6)
Most recent HbA_1c_, mean (SD), %	8.1 (1.7)	8.2 (1.9)	8.1 (1.8)
Use of any diabetes medication	75 (85)	67 (77)	142 (81)
How many years ago you were told you have diabetes, mean (SD)	21 (12)	20 (12)	20 (12)
Peripheral neuropathy	62 (71)	63 (72)	125 (72)
Severe PAD by ABI	4 (5)	7 (8)	11 (6)
Most recent creatinine, median (IQR), mg/dL	1.2 (1.0-1.6)	1.2 (0.9-1.7)	1.2 (0.9-1.7)
Dialysis dependent	12 (14)	12 (14)	24 (14)
Retinopathy	32 (36)	26 (30)	58 (33)
Foot conditions			
Any healed previous foot complication	48 (55)	43 (49)	91 (52)
Previous foot ulcer or wound	46 (52)	38 (44)	84 (48)
Previous major foot infection	22 (25)	24 (28)	46 (26)
If yes, did it involve osteomyelitis	14 (67)	16 (67)	30 (67)
Previous major foot surgery	21 (24)	19 (22)	40 (23)
Months from healing of most recent foot complication to randomization, median (IQR)^b^	13 (5-40)	7 (2-27)	13 (3-36)
Months from healing of most recent foot complication to randomization^b^			
<3	10 (21)	11 (26)	21 (23)
3 to <12	9 (19)	13 (30)	22 (24)
12 to <36	13 (27)	10 (23)	23 (25)
≥36	16 (33)	9 (21)	25 (27)
Current foot ulcer or wound(s)	8 (9)	7 (8)	15 (9)
Current preulcer lesion	11 (13)	6 (7)	17 (10)
High arch	10 (11)	9 (10)	19 (11)
Dropped foot	21 (24)	11 (13)	32 (18)
Hammer or claw toe	67 (76)	57 (66)	124 (71)
Bunion	7 (8)	6 (6)	12 (7)
Onychomycosis	75 (85)	75 (86)	150 (86)
Charcot deformity	5 (6)	2 (2)	7 (4)
Tinea pedis	0	1 (1)	1 (1)
Lower extremity edema	28 (33)	22 (25)	50 (29)
Foot care			
Wear special shoes for diabetes	53 (60)	41 (47)	94 (54)
In the past 4 weeks, how often have you…			
Washed your feet with soap and water			
Not at all	1 (1)	0	1 (1)
About once or twice each month	1 (1)	0	1 (1)
Once each week	6 (8)	7 (9)	13 (7)
Several times each week	27 (34)	31 (40)	58 (33)
Daily	44 (56)	39 (51)	83 (47)
Washed your feet with a washcloth			
Not at all	12 (15)	13 (17)	25 (16)
About once or twice each month	0	4 (5)	4 (4)
Once each week	4 (5)	4 (5)	8 (5)
Several times each week	22 (28)	16 (21)	38 (24)
Daily	41 (52)	40 (52)	81 (52)
Used lotion on your feet			
Not at all	23 (26)	22 (25)	45 (26)
About once or twice each month	3 (3)	3 (3)	6 (3)
Once each week	9 (10)	11 (13)	20 (11)
Several times each week	16 (18)	13 (15)	29 (17)
Daily	37 (42)	38 (44)	75 (43)
Comorbidities			
Hypertension	82 (93)	80 (92)	162 (93)
Hyperlipidemia	73 (83)	67 (77)	140 (80)
Most recent LDL cholesterol, median (IQR), mg/dL	80 (60-98)	68 (51-89)	76 (56-94)
Most recent HDL cholesterol, median (IQR), mg/dL	42 (36-48)	44 (34-54)	43 (35-50)
History of CVA or TIA	5 (6)	8 (9)	13 (7)
Coronary artery disease	21 (24)	19 (22)	40 (23)
Congestive heart failure	11 (13)	10 (11)	21 (12)
Chronic venous insufficiency	31 (35)	19 (22)	50 (29)
Current smoking	18 (20)	12 (14)	30 (17)
Current depression	20 (23)	20 (23)	40 (23)
Current schizophrenia	2 (2)	3 (3)	5 (3)
Current bipolar disorder	2 (2)	3 (3)	5 (3)
History of substance use	19 (22)	13 (15)	32 (18)
History of homelessness	9 (10)	7 (8)	16 (9)

Abbreviations: ABI, Arterial Brachial Index; BMI, body mass index (calculated as weight in kilograms divided by height in meters squared); CVA, cerebrovascular accident; HbA_1c_, hemoglobin A_1c_; HDL, high-density lipoprotein; LDL, low-density lipoprotein; PAD, peripheral artery disease; TIA, transient ischemic attack.

SI conversion factors: To convert creatinine to micromoles per liter, multiply by 88.4; LDL and HDL cholesterol to millimoles per liter, multiply by 0.0259; and to convert HbA_1c_ to proportion of total hemglobin, multiply by 0.01.

^a^ Race and ethnicity were obtained from participants’ medical records.

^b^ Values available only for participants with a healed previous foot complication.

### Primary Outcome

Twenty-six of the 175 participants (15%) had a new foot complication (new chronic foot ulcer, moderate to severe foot infection, or foot amputation from a new ulcer). Median (IQR) time from randomization to development of the new foot complication was 232 (115-315) days, with a range of 5 to 348 days. Twenty-one of 66 participants (32%) with a history of a foot ulcer within the past 36 months had a new foot complication. Four of 26 participants (15%) with a new foot complication had moderate to severe PAD, as measured by the Arterial Brachial Index.

**Figure 2.**
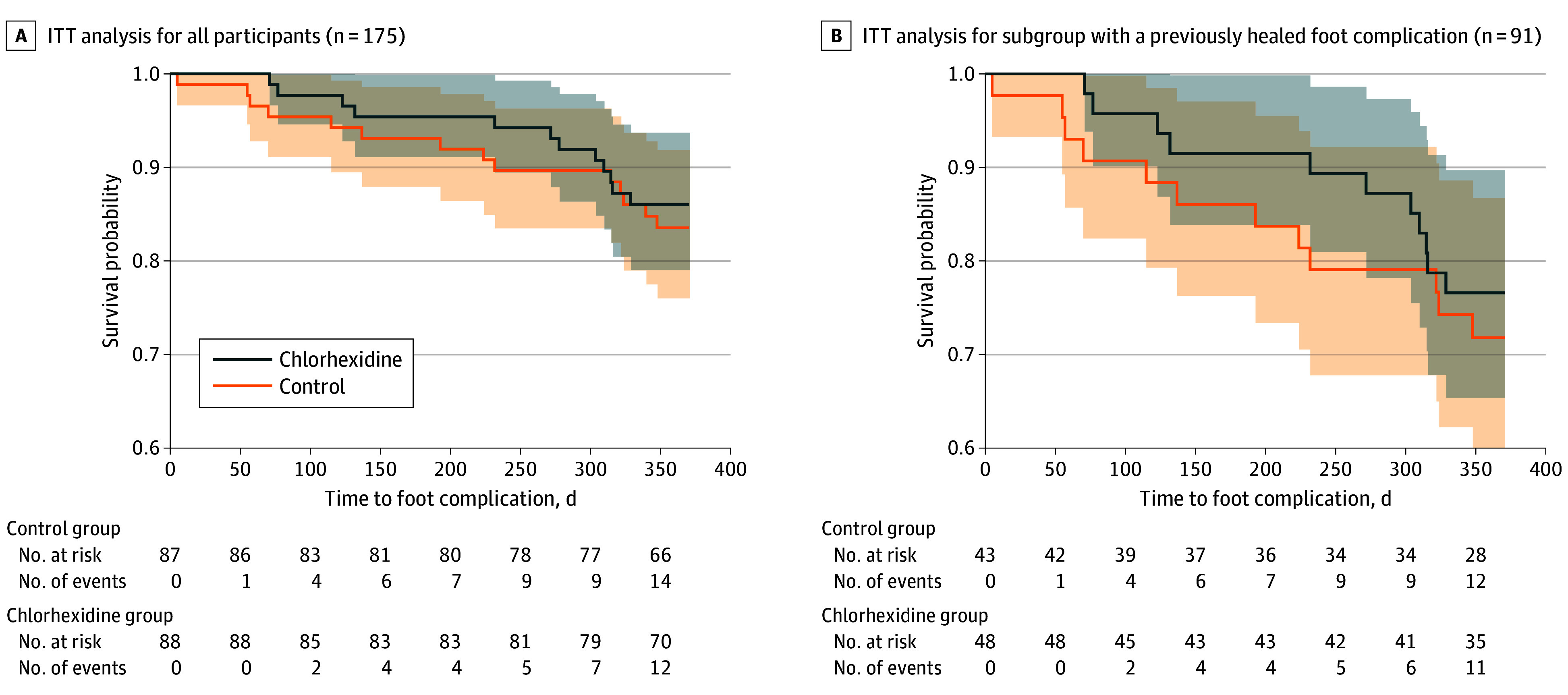
Time to Foot Complication by Treatment Group Shading represents 95% CIs. ITT indicates intention to treat.

Twelve of 88 participants (14%) in the chlorhexidine group and 14 of 87 participants (16%) in the control group developed a new foot complication. In an ITT analysis among all participants, the HR comparing the risk of a new foot complication between the chlorhexidine group and the control group was 0.83 (95% CI, 0.39-1.80) (Figure 2A). The log-rank test comparing the distribution of time to outcome between the 2 groups was not significant (*P* = .64). After adjusting for the presence of a current preulcer foot lesion at randomization, the HR was 0.75 (95% CI, 0.35-1.64). If participants with severe PAD were excluded from the adjusted analysis, the HR was 0.86 (95% CI, 0.37-2.00).

Of the 12 participants in the chlorhexidine group with a new foot complication, 8 (67%) developed a new chronic foot ulcer, 3 (25%) developed an infection from a new acute foot ulcer, and 1 (8%) needed an amputation for a new acute foot ulcer. Of the 14 participants in the control group with a new foot complication, 13 (93%) developed a new chronic foot ulcer and 1 (7%) needed an amputation for a new acute foot ulcer. Of the 24 participants who developed a chronic foot ulcer or infection from a new acute ulcer, 9 (38%) went on to have a full or partial foot amputation in the 12 months after outcome diagnosis.

The prespecified subgroup included 91 participants with a resolved foot complication prior to randomization (eMethods in Supplement 2). The test for a difference in treatment effect between participants with and without a resolved prior foot complication was not significant (β = 0.35, SE = 1.29; *P* = .79). In an ITT analysis among this subgroup, the HR between the chlorhexidine group and the control group was 0.78 (95% CI, 0.34-1.77) (Figure 2B). After adjusting for the presence of a current preulcer foot lesion at randomization and months since healing of a previous foot complication, the HR was 0.82 (95% CI, 0.36-1.88). If participants with severe PAD were excluded from the adjusted analysis, the HR was 0.96 (95% CI, 0.38-2.39).

### Secondary Outcome

**Figure 3.**
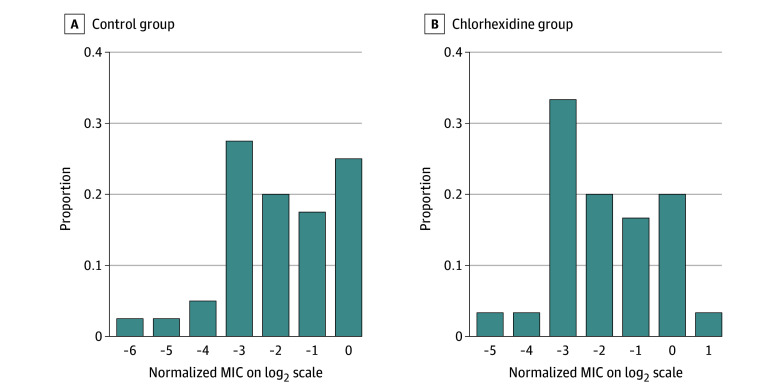
Distribution of Normalized Chlorhexidine Minimum Inhibitory Concentration (MIC) Values for ESKAPE and Other Diabetic Foot Infection (DFI) Pathogens on Feet Approximately 4 Weeks After Stopping the Intervention by Treatment Group ESKAPE pathogens are *Enterobacter* spp, *Staphylococcus aureus*, *Klebsiella pneumoniae*, *Acinetobacter baumannii*, *Pseudomonas aeruginosa*, and *Enterococcus faecium*. DFI pathogens are *Escherichia coli*, *Enterococcus faecalis*, and *Streptococcus* spp.

Of the 141 participants who had a culture approximately 4 weeks after stopping the intervention, 55 (39%) were colonized with at least 1 ESKAPE or other DFI pathogen and 86 (61%) were not colonized (Table 2). There was a total of 30 ESKAPE and other DFI pathogens from the chlorhexidine group (mean [SD] normalized MIC, −1.83 [1.44]) compared with 40 from the control group (mean [SD] normalized MIC, −1.88 [1.52]). The distribution of normalized MIC to chlorhexidine by treatment group is shown in Figure 3. Normalized MICs to chlorhexidine among bacterial pathogens detected on the feet were not significantly different between treatment arms (mean difference, −0.04; 95% CI, −0.75 to 0.67).

**Table 2.** Colonization of the Feet by Bacterial Pathogens After the Intervention by Treatment Group

ESKAPE and DFI pathogens	Participants, No. (%)	
	Chlorhexidine group (n = 71)	Control group (n = 70)
Total not colonized	44 (62)	42 (60)
Total colonized with any pathogen	27 (38)	28 (40)
* Acinetobacter baumannii*	0	2 (3)
*Enterobacter cloacae* complex	0	3 (4)
* Enterococcus faecalis*	7 (10)	11 (16)
* Enterococcus faecium*	2 (3)	4 (6)
* Escherichia coli*	1 (1)	2 (3)
* Klebsiella pneumoniae*	5 (7)	4 (6)
* Pseudomonas aeruginosa*	5 (7)	5 (7)
* Staphylococcus aureus*	10 (14)	9 (13)
* Streptococcus *spp	0	0

Abbreviations: DFI, diabetic foot infection (pathogens: *Escherichia coli*, *Enterococcus faecalis*, and *Streptococcus *spp); ESKAPE, *Enterobacter* spp, *Staphylococcus aureus*, *Klebsiella pneumoniae*, *Acinetobacter baumannii*, *Pseudomonas aeruginosa*, and *Enterococcus faecium*.

### Adverse Events and Adherence

There were 60 adverse events during the study. Thirty-three adverse events (55%) occurred in the chlorhexidine group, 26 (43%) occurred in the control group, and 1 (2%) occurred to a participant who was enrolled but was never randomly assigned to a treatment group. No adverse events were related to the study products or procedures. No adverse events involving dermatological changes were reported. Four participants died during the study, but none of these deaths were deemed related to the study. The intervention was well tolerated: 145 participants (83%) continued it over the study period.

Seventy-seven percent (n = 134) of all participants used at least 80% of the wipes during their time at risk for a new foot complication. There was no difference between treatment arms in the proportion of participants using at least 80% of the wipes. Median (IQR) wipe use was 99% (80%-100%) of expected wipes. All foot swabs that were tested for the presence of chlorhexidine were negative for the control group. Many foot swabs (100 of 212 [47%]) that were tested for the presence of chlorhexidine were positive for the chlorhexidine group.

### Participant Feedback on Intervention

At the end of their time in the study, 142 participants (81%) provided feedback on daily use of the wipes and lotion. Of these 142 participants, nearly all (141 [99%]) shared positive feedback, mostly related to their feet feeling soft, clean, and smooth and how they enjoyed the routine of cleaning their feet daily. Some participants (30 [21%]) also had negative feedback, mostly related to the wipes making their feet feel tingly, dry, or sticky and how they did not like needing to have a daily routine.

## Discussion

Daily use of a chlorhexidine wipe for foot washing compared with a soap and water wipe did not lead to a statistically significant reduction in the hazard of new foot complications among veterans at high risk for a diabetic foot ulcer. When significant imbalances in key variables were adjusted for, there was little change. We chose to adjust for preulcer at randomization because of the imbalance with randomization and its association with the primary outcome. A preulcer is often the precursor of an ulcer.^3^ In the prespecified subgroup sensitivity analyses of those with a prior foot complication, we also adjusted for time since healing for the same reasons.^3^ These sensitivity analyses made little difference to effect estimates and no difference to conclusions regarding the primary outcome. Chlorhexidine resistance, as estimated by chlorhexidine MICs from bacteria on the feet after trial completion, did not differ significantly between treatment groups, and the bounds of the CIs were not clinically significant and consistent with the error of the assay (ie, an absolute log_2_–normalized mean difference MIC less than 1 or a less than 2-fold difference). Adverse events were not significantly greater in the chlorhexidine group.

### Comparison to Other Studies

To our knowledge, there are no other studies on the efficacy of foot washing with a chlorhexidine wipe or a soap and water wipe to decrease foot complications in veterans with diabetes. Daily foot washing with soap and water is the recommended standard of care for people with diabetes.^18^ Chlorhexidine is used to decolonize people with *S aureus* colonization to reduce infections, although the RCT demonstrating this finding had a comparator group that used education as opposed to a hygiene intervention other than chlorhexidine.^13^ This RCT used soap and water wipes to represent the standard of care for the control arm because of the ability to blind participants to their treatment. We may have provided a similar level of microbial burden reduction with the control arm compared with the chlorhexidine arm. Chlorhexidine resistance, as estimated by chlorhexidine MICs from bacteria on the feet after trial completion, did not differ significantly between groups. There are no established MIC cut points for chlorhexidine, a topical antiseptic widely used in health care. Because of chlorhexidine’s wide use, there is concern that its MICs could increase over time and lead to a decrease in its antimicrobial activity.^19^ Therefore, any reported difference would be of concern. Results of this trial are congruent with those of the REDUCE-MRSA (Randomized Evaluation of Decolonization vs Universal Clearance to Eliminate Methicillin-Resistant *Staphylococcus aureus*) trial, which assessed chlorhexidine susceptibility of methicillin-resistant *S aureus* (MRSA) isolates.^20^ Lower susceptibility to chlorhexidine and carriage of *qacA* or *qacB* gene were rare among 3173 MRSA isolates in the REDUCE-MRSA trial.

Adherence to wipe use was relatively high across both arms, with 77% of all participants reporting using at least 80% of the wipes. This finding indicates an increase in foot hygiene compared with reported foot hygiene before enrollment, at which 52% of all participants reported using a washcloth to clean their feet daily. We detected chlorhexidine on the feet of those receiving chlorhexidine wipes only 47% of the time. Chlorhexidine levels on the skin decrease over time but are typically still detectable within 24 hours of use.^21^ This fact suggests that adherence may have been lower than reported by participants. It is unclear how often chlorhexidine needs to be used in order to decrease foot complications. We know from inpatient studies that microbial burden rebounds within 24 hours; however, RCTs that demonstrated a decrease in MRSA infections and hospitalizations due to infection used chlorhexidine less frequently—twice a week^11^ or 10 days a month.^13^

### Strengths and Limitations

To our knowledge, this was the largest clinical trial on preventing diabetic foot ulcers in medical literature, which is a strength of this trial. The trial also has several limitations. First, the study population was nearly all males and predominantly Black or African American; thus, we do not know whether the findings are generalizable to the general population. Second, the trial has multiple lessons for future clinical trials. The eligibility criteria were too broad. Veterans with a history of a foot ulcer within the past 36 months had almost all of the outcomes, with 32% of them developing a new foot complication. Only 15% of the participants developed a new foot complication, which was much lower than the 40% we anticipated in the control group, most likely because we were unable to exclusively enroll participants with a recently healed foot ulcer. Additionally, we did not exclude veterans with moderate to severe PAD, which led to new foot complications that were primarily ischemic and thus unlikely to be prevented by the intervention. Six percent of the population had moderate to severe PAD, as measured by the Arterial Brachial Index, and accounted for 15% of the new foot complications. Future clinical trials on preventing ulcer recurrence should focus on people with diabetes who have a history of a healed plantar ulcer without evidence of severe PAD.

## Conclusions

In this RCT, we did not detect a statistically significant decrease in the risk of new foot complications after daily use of chlorhexidine wipes on the feet for 1 year compared with daily use of soap and water wipes among veterans with diabetes. Based on these results, it is unclear whether daily use of chlorhexidine on the feet is effective at reducing new foot complications compared with daily use of soap and water. Better interventions are needed to prevent the recurrence of foot ulcers in people with diabetes. Structured daily foot washing with chlorhexidine or soap-and-water wipes was well tolerated by the participants. Future studies on diabetic foot ulcer prevention should incorporate the lessons learned in this trial to assess whether actively promoting daily foot hygiene could reduce ulcer recurrence in those with a history of foot complication.

## Notes

Supplement 1.
Trial Protocol and Statistical Analysis Plan

Supplement 2.
eMethods.
**eTable.** Chlorhexidine MIC_50_s From the Literature
eReferences

Supplement 3.
Data Sharing Statement
