# Error in Figure 3

**DOI:** 10.1001/jamanetworkopen.2025.4939

**Published:** 2025-03-11

In the Original Investigation titled “Chlorhexidine vs Routine Foot Washing to Prevent Diabetic Foot Ulcers: A Randomized Clinical Trial,”^1^ published online February 18, 2025, the x-axis of Figure 3B was labeled from –6 to 0 but should have been labeled from –5 to 1. This article has been corrected.
