# Supplementary material for: Chlorhexidine vs Routine Foot Washing to Prevent Diabetic Foot Ulcers: A Randomized Clinical Trial
Source: JAMA Netw Open. 2025 Feb 18;8(2):e2460087. doi: 10.1001/jamanetworkopen.2024.60087 (PMC11836759; doi:10.1001/jamanetworkopen.2024.60087)
Supplement: Supplement 1. — Trial Protocol and Statistical Analysis Plan [file jamanetwopen-e2460087-s001.pdf]

PROTOCOL NAME
Grant Title: Preventing Diabetic Foot Ulcers through Manipulating the Skin Microbiota
Short title: Preventing Diabetic Foot Ulcers through Cleaner Feet

CICERO protocol number: HP-79894
Modification #: 22
Version #: 15.0
PROTOCOL VERSION DATE: 1/25/2023

Commonly Used Abbreviations

S. aureus	*Staphylococcus aureus*
VAMHCS	Veterans Affairs Maryland Health Care System
PAVE	Prevention of Amputation in Veterans Everywhere
R&D	Research and Development
VHA	Veterans Health Administration
UMB	University of Maryland Baltimore
IRB	Institutional Review Board

GENERAL INFORMATION

Name and address of the study monitor and person authorized to sign the protocol and amendments:

Mary-Claire Roghmann, MD, MS
Infectious Disease Staff Physician and Research Health Scientist
Head, Epidemiology Section
Medical Care Clinical Center
VA Maryland Health Care System

Professor of Epidemiology and Public Health and Medicine
University of Maryland School of Medicine
10 S. Pine St., MSTF 3-36
Baltimore, MD 21201
Phone (410) 706-0062
Fax (410) 706-0098
E-mails mroghmann@som.umaryland.edu
Mary-Claire.Roghmann@va.gov

Name and addresses of the laboratories, study sites and/or other institutions involved in the study:

Laboratories-
Baltimore VA Medical Center
Microbiology Research Lab, 3C-112
10 North Greene Street
Baltimore, MD 21201

Microbiology Research Lab
Department of Pathology
Allied Health Building, Room 401 and 450
100 Penn Street
Baltimore, MD 21201

Participant Recruitment Sites-
Baltimore VA Medical Center
Outpatient Clinics
10 North Greene Street
Baltimore, MD 21201

Loch Raven VA Community based Outpatient Clinics
3900 Loch Raven Boulevard
Baltimore, MD 21218

Perry Point VA Medical Center
Outpatient Clinics
Perry Point, MD 21902

Study form storage-
University of Maryland School of Medicine
MSTF 360
10 South Pine Street
Baltimore, MD 21201

Clinical visits-
VA Medical Healthcare Centers

Revision History-Modifications made within the protocol and/or consent

**Protocol Version**	**Date**	**Amendments**
V 15.0	01/25/2023	1. Modified study variables in Appendix to accurately reflect what was collected during the study.<br>2. Removed specific antibiotics we are testing
V 14.0	07/06/2022	1. Changed the time in the study to be 15 months and not 13 months.<br>2. Added organisms we are culturing swabs for
V 13.0	8/12/2020	1. The V7 visit was changed to after 2 weeks instead of 2-6 weeks<br>2. In the ICF, it was added that photos will only be used to assess foot health.
V 12.0	6/30/2020	1. Combined the ICF and HIPAA in one document<br>2. Added the ability to consent remotely<br>3. Editorial changes to be consistent with other documents<br>4. Added that one bag per participant kit will be kept to make sure the correct wipe was distributed to the participant
V 11.0	5/18/2020	1. Revised sites for clinical visits to be anywhere in the VAMHCS<br>2. Added ability to pay participants with a gift card<br>3. Added participants that are having remote visits will self-swab their feet and take photos of their feet.<br>4. Added instructions on how to take foot swabs.<br>5. Added instructions on how to take foot photos<br>6. Editorial changes to be consistent with other documents
V10.0	03/11/2020	1. Made edits to the protocol to be consistent with the informed consent document<br>2. Edited to remove nasal swabs if participant exhibits respiratory symptoms<br>3. Edited to make changes to study visits related to COVID-19
V9.0	02/06/2020	1. Modified when visit 7 could occur
V8.0	9/26/2019	2. Added language related to transfer of data to and from CRISP
V7.0	8/27/2019	1. Modified the eligibility criteria<br>2. Added language to include a final visit for participants who were randomized and were withdrawn from the study.<br>3. Updated the definition for study primary/secondary endpoint

		4. We added an informational flyer for VA providers as a way of providers to refer patients that may be eligible for the study.<br>5. We updated our flyer and introductory letter for veterans with the new eligibility criteria
V6.0	4/29/2019	1. Modified the eligibility criteria<br>2. Added language to include the option of receiving weekly medication reminder texts through the Annie App instead of from a cell phone.<br>3. We added the ability to follow-up with participants if they are eligible, interested in the study, but want to review the protocol at home first
V5.0		1. Modified the eligibility criteria<br>2. We changed the outcome definition to indicate that we are aiming to prevent new foot complications.<br>3. Modified the study variables that are being collected.
V4.0	12/19/2018	1. Added language to withdraw participant if they reach the end-point.<br>2. Modified the eligibility criteria<br>3. Adding a 7th skin site on the foot and an 8th swab for chlorhexidine testing<br>4. Made changes to the initial interaction with potential participant.<br>5. Modified the study variables that are being collected.
V3.0	9/21/2018	1. Removed monthly sampling of feet and mailing of specimens<br>2. Added photography of feet<br>3. Added language to be clearer what was being done at each visit<br>4. Added language to include monthly calls about health and weekly texts/calls as medication reminders<br>5. Added to participant responsibilities: respond to contact from staff<br>6. Increased compensation to $50 per visit and removed language about compensation for monthly self-swabs. Total compensation = $350<br>7. Added language about CRISP<br>8. Added clinicaltrials.gov # and type of clinical trial<br>9. Modified the eligibility criteria<br>10. Added language about when the eligibility criteria needed to be signed<br>11. Added the information about an introductory letter and opt-out procedures<br>12. Study cloths will be repackaged with seven cloths per package instead of one

		13. Added more detail about signs of intolerance<br>14. Added the ability to use VINCI<br>15. Modified the Data Collection, Handling and Storage section to reflect the collection of full SSNs, not just the last 4 digits.<br>16. Modified the analysis plan and sample size calculations<br>17. Increased the sample size in order to compensate for potential withdrawal of study participants.<br>18. Modified language related to repackaging and storage of study product to indicate that study staff will be repackaging wipes and study product will be stored outside of the VA investigational pharmacy.
V2.0	5/17/2018	No changes to the protocol. The following was modified within the IRB protocol and HIPAA<br>1. Additional questions were addressed within CICERO<br>2. The IRB was updated with the ClinicalTrials.gov number.<br>3. A version date was added to the HIPAA document
V1.0	5/14/2018	N/A – Original Protocol

Table of Contents

PROTOCOL SUMMARY **7**

LAY SUMMARY **7**

1 BACKGROUND INFORMATION AND RATIONALE **8**

1.1 BACKGROUND AND RATIONALE 8
1.2 RISK/BENEFITS 8
1.3 STUDY CONDUCT 9

2 STUDY OBJECTIVES **9**

3 STUDY DESIGN **10**

3.1 PRIMARY STUDY ENDPOINTS/SECONDARY ENDPOINTS 10

4 STUDY DESIGN/TYPE **10**

4.1 DURATION 10

5 SELECTION AND WITHDRAWAL OF PARTICIPANTS **10**

5.1 SELECTION OF STUDY POPULATION 10
5.2 INCLUSION CRITERIA 10
5.3 EXCLUSION CRITERIA 11
5.4 PARTICIPANT WITHDRAWAL 11

6 INFORMED CONSENT PROCESS **11**

6.1 RECRUITMENT 11
6.1.1 Distribution of IRB approved informational flyer *12*
6.1.2 Mailing of an Introductory letter and an opt out card *12*
6.2 INFORMED CONSENT 13

7 STUDY INTERVENTION **13**

7.1 STUDY PRODUCT DESCRIPTION 13
7.1.1 Acquisition *13*
7.1.2 Formulation, Packaging, and Labeling *14*
7.1.3 Product Storage and Stability *14*
7.2 DOSAGE, PREPARATION AND ADMINISTRATION OF STUDY INTERVENTION 14
7.3 MODIFICATION OF STUDY INTERVENTION 14
7.4 ACCOUNTABILITY PROCEDURES FOR THE STUDY INTERVENTION 14
7.5 ASSESSMENT OF PARTICIPANT COMPLIANCE WITH STUDY INTERVENTION 14

8 STUDY PROCEDURES/EVALUATIONS **15**

8.1 STUDY VISITS 16
8.2 LABORATORY EVALUATIONS 17
8.3 CLINICAL EVALUATIONS 17

9 UNANTICIPATED PROBLEMS AND SERIOUS ADVERSE EVENTS **17**

10 QUALITY CONTROL AND QUALITY ASSURANCE **17**

11 STATISTICAL PLAN **17**

11.1 ANALYSIS PLAN 17
11.1 SAMPLE SIZE CALCULATION 18

12 ETHICAL CONSIDERATIONS **18**

13 DATA COLLECTION, HANDLING AND STORAGE **18**

14 VA INFORMATICS AND COMPUTING INFRASTRUCTURE (VINCI) **19**

15 CHESAPEAKE REGIONAL INFORMATION SYSTEM FOR OUR PATIENTS (CRISP) **22**

Protocol Summary

Population: Up to 200 veterans at risk for diabetic foot ulcers

Site: VA Maryland Health Care System (VAMHCS)

Study Duration: Approximately 5 years

Clinical Trial: Phase IIb

Clinicaltrials.gov #: NCT03503370

IND exempt

Study Design: Randomized double-blind clinical trial comparing a) a daily foot care regimen with cloths containing 2% chlorhexidine to b) a daily foot care regimen with cloths not containing 2% chlorhexidine

Objectives:
Primary: To determine if chlorhexidine reduces foot complications including chronic foot ulcer, foot infection or foot amputation.
Secondary: To determine if chlorhexidine increases antibiotic resistance among bacterial pathogens on feet.
Exploratory: To describe changes in the microbiota of the feet with chlorhexidine and foot complications

Treatment Regimens: SAGE 2% Chlorhexidine Gluconate Cloths versus SAGE Comfort Bath Cloths

Route of Administration: Topical application on the feet

Dose and Interval: 1 cloth daily

Duration of Participant's Participation: Up to 15 months

Lay Summary
Foot complications are among the most serious and costly complications of diabetes. People with diabetes have a 10-fold increased risk for a leg or foot amputation compared to those that do not have diabetes. Amputation of all or part of foot is usually preceded by a foot ulcer, which became infected. This is a clinical trial to test the effectiveness of a topical antiseptic, chlorhexidine, for daily foot cleaning on reducing new foot complications including chronic foot ulcer or wound, foot infection, or foot amputation in Veterans at high risk for a foot ulcer.

Keywords: Foot ulcer, diabetes, topical chlorhexidine, prevention, clinical trial

1 Background Information and Rationale

1.1 Background and Rationale

Between 10 and 25% of people with diabetes will develop a foot ulcer during their lifetime. Foot ulcers are a leading cause of hospitalization in people with diabetes and the primary cause of non-traumatic amputation. About 40% of people with diabetes with a recently resolved foot ulcer will have a recurrence within one year despite current prevention efforts. Thus the development of new interventions to decrease the risk of foot ulcers in high risk people is urgently needed. The prevalence of diabetes in the Veterans Health Administration's patient population is about 24% making this a priority clinical issue for veterans' care.

A foot ulcer typically begins with minor trauma to the foot in a patient with diabetic neuropathy. The minor skin trauma fails to heal resulting in a skin ulcer. The ulcer leads to infection, particularly due to *Staphylococcus aureus*, and often to gangrene resulting in an amputation. The role of the skin microbiota in the development of foot ulcers is unknown. Multiple skin disorders and infections are hypothesized to result from imbalance in the microbiota and the host inflammatory and immune responses. Impaired wound healing is hypothesized to result in part from dysregulated inflammation and microbial colonization suggesting a possible mechanism by which the skin microbiota could promote faulty wound healing after a minor trauma leading to a foot ulcer.

Prior work has shown that the feet of diabetic veterans have an increased *S. aureus* load compared with non-diabetic veterans. Our preliminary data suggest that there is an increased S. aureus and total bacterial load on the feet of diabetic veterans at high risk for future foot ulcer compared to diabetic veterans at low risk of a future foot ulcer. We also found a higher relative abundance of staphylococci in the skin microbiota on the heels of people with diabetes at high risk for foot ulcers compared to those at low risk. Thus our data suggest that there are absolute differences in bacterial load and perhaps relative differences in abundance of bacterial taxa on the feet which could be part of the causal pathway for developing a foot ulcer. If so, altering the skin microbiota of the feet could reduce the risk of foot ulcers.

Chlorhexidine is a potential intervention to decrease the risk of diabetic foot ulcer through reducing the bacterial and fungal load including skin pathogens such as *S. aureus*. It is a broad-spectrum antiseptic agent with an excellent safety profile. It prevents healthcare associated infections, such as surgical site infection, after iatrogenic skin breakdown through reducing the microbial load on the skin which has similarities to the pathogenesis of diabetic foot ulcers. To explore the effect of chlorhexidine on the skin microbiota of the foot, we had Veterans with diabetes at high risk for foot ulcer use chlorhexidine on their feet. We found an absolute decrease in *S. aureus* and overall bacterial load and a decrease in the relative abundance of staphylococci among the foot microbiota. Thus we propose the following study to test the effect of chlorhexidine reduces new diabetic foot ulcers.

1.2 Risk/Benefits

The risks of the proposed study are minimal. There are possible risks and discomforts from this study for participants.

SAGE® 2% CHLORHEXIDINE GLUCONATE* CLOTH is an FDA- approved formulation and application for topical 2% chlorhexidine, an antiseptic solution which is available without a prescription. It is widely used for pre-operative skin cleansing and extremely well tolerated. Mild skin irritation can occur; this stops after chlorhexidine is stopped. A systemic allergic reaction including low blood pressure can occur; this is very rare.

SAGE® COMFORT BATH CLOTHS is a cloth soaked in mild, rinse-free cleansers and skin-protecting moisturizers like aloe and vitamin E, which is available without a prescription. It is widely used for bathing hospitalized patients and extremely well tolerated. An allergic reaction could occur; this is very rare.

The sampling methods for the anterior nares and skin sites is physically non-invasive, but there could be some discomfort during the procedure. The potential discomfort from obtaining the specimens does not place any participant at physical risk. A trained research coordinator will use very gentle pressure while obtaining the specimen to minimize these risks.

There is a risk that health information could be in accidentally disclosed to others outside the study. There is a potential for breach of privacy. Privacy and Confidentiality will be protected to the extent permitted by law.

There may be adverse events that are not yet known.

There is no alternative treatment or procedure to participating in this study, but individuals have the right to decline participation in this research without any prejudice to their care or benefits.

There are potential direct benefits to study participants. Participants will receive education on foot self-care. Participants receiving either regimen will have cleaner feet. This study will provide knowledge to determine whether topical chlorhexidine might be used to prevent diabetic foot ulcers and their complications.

1.3 Study Conduct

This study will be conducted in compliance with the protocol approved by the UMB Institutional Review Board (hereafter referred to as IRB) and VAMHCS R&D Committee and according to Good Clinical Practice standards and the VHA Handbook 1200.05 Requirements for the Protection of Human Subjects in Research. No deviation from the protocol will be knowingly implemented without the prior review and approval of the IRB except where it may be necessary to eliminate an immediate hazard to a research participant. In such case, the deviation will be reported to the IRB as soon as possible.

2 Study Objectives

Primary: To determine if chlorhexidine reduces new foot complications including chronic foot ulcer, foot infection or foot amputation.
Secondary: To determine if chlorhexidine increases antibiotic resistance among bacterial pathogens on feet.
Exploratory: To describe changes in the microbiota of the feet with chlorhexidine and foot complications.

3 Study Design

3.1 Primary Study Endpoints/Secondary Endpoints

Foot complications. We will assess participants for new foot complications to include: new chronic foot ulcers or foot infections or foot amputations on a monthly basis. Our primary outcome is time from randomization until either 1) a new chronic (present 28 days from initial diagnosis) foot ulcer or wound or 2) a moderate or severe foot infection (as defined IDSA Diabetic Foot Infection Severity classification: Table 2[1]) not from an existing ulcer or 3) a foot amputation for a new ulcer.

Susceptibility to chlorhexidine and other antibiotics among bacterial pathogens. Participants will have cultures performed with a nylon flocked swab at enrollment and at 13 months approximately 4 weeks after stopping the intervention.

4 Study Design/Type

This is a multi-center, randomized, double blind (participant, outcome assessor), parallel group clinical trial comparing daily foot care with cloths containing 2% chlorhexidine compared to daily foot care with cloths not containing chlorhexidine.

4.1 Duration

Study participants will have study visits over a period of up to 15 months.

5 Selection and Withdrawal of Participants

5.1 Selection of Study population

All study participants will be recruited from the outpatient population in the VA Maryland Health Care System. Some eligibility criteria cannot be assessed on the date of consent. Therefore, the eligibility checklist can be signed any time before the date of randomization.

5.2 Inclusion Criteria

- Adults >= 18 years
- Clinical diagnosis of diabetes
- At risk for a new diabetic foot ulcer due to:
  1) Past history of a diabetic foot ulcer or
  2) Past history of major foot surgery including partial foot amputation (e.g. toe) or
  3) Past history of major foot infection or
  4) Neuropathy and onychomycosis and hemoglobin A1C >8% or
  5) Neuropathy and peripheral vascular disease or
  6) Dialysis or
  7) Past history of Charcot foot or
  8) Past history of peripheral vascular surgery or angiography with stent
- Two feet (can have amputations of part of the foot)
- At least one foot without a foot ulcer

---

[1] Benjamin A. Lipsky, Anthony R. Berendt, Paul B. Cornia, James C. Pile, Edgar J. G. Peters, David G. Armstrong, H. Gunner Deery, John M. Embil, Warren S. Joseph, Adolf W. Karchmer, Michael S. Pinzur, Eric Senneville, 2012 Infectious Diseases Society of America Clinical Practice Guideline for the Diagnosis and Treatment of Diabetic Foot Infections, *Clinical Infectious Diseases*, Volume 54, Issue 12, 15 June 2012, Pages e132–e173, https://doi.org/10.1093/cid/cis346

- Permanent mailing address suitable for provision of specimen collection materials and telephone suitable for up to weekly follow-up
- Able to give written informed consent

5.3 Exclusion Criteria

- Amputation of the foot planned to treat current foot ulcer or wound
- Current foot infection
- Use of topical chlorhexidine on feet 7 days prior to randomization
- History of an allergic reaction to chlorhexidine
- Unable to use wipes for foot care
- Inability to walk
- Life expectancy less than 12 months
- Plans to move out of the area in the next 13 months
- Requires equivalent of institutional care (e.g. nursing home)
- Any other criteria which, in the investigator's opinion, would compromise the safety of the study, the ability of a subject to participate, or the results of the study.

5.4 Participant Withdrawal

Study participants will be withdrawn from the study if found to have been initially ineligible or intolerant of the study procedures. Study participants who have missed two consecutive study visits will no longer be contacted regarding additional study visits and will not receive additional study intervention products (wipes and swabs). Study participants whose study visits are re-scheduled as part of the safety concerns related to COVID-19 will still be contacted regarding the study and will still receive study supplies if they indicate they would like to still be in the study. However, study participants who have missed two consecutive visits or have been randomized to the study intervention and then withdrawn from the study can contact study staff and return for a visit #7 (outlined below) at any point prior to 1-year post randomization. This visit #7 should be performed at least 2 weeks after the participant stops taking study medication. Study staff will continue to perform medical record review for foot ulcers, infection or amputation and record clinical management of ulcers, infection or amputation for withdrawn participants unless the participant specifically requests otherwise.

Study participants will also be withdrawn if they reach the primary study endpoint before 13-months.

6 Informed Consent Process

6.1 Recruitment

We are requesting a partial HIPAA waiver for recruitment to allow study staff to review administrative data from the VAMCHS Podiatry Service and then the medical record of potential participants to determine initial eligibility. Study staff will review and document the administrative data and then medical records of potential participants to determine if they meet eligibility criteria. Accessing this information from administrative data and the participant's medical record reduces the burden on the participant with regard to time and research procedures, increases the feasibility of the study, and confers no more than minimal, additional risk.

All study participants will be recruited from the VA Maryland Health Care System. They will be identified by administrative codes used for Prevention of Amputation in Veterans Everywhere (PAVE) initiative or by using the VA Informatics and Computing Infrastructure (VINCI). PAVE is a VA directive which requires VA medical centers to track Veterans by their risk of future lower extremity amputation. VINCI can provide lists of veterans that meet eligibility criteria using data from the electronic medical record. We will use the PAVE administrative codes and data from VINCI to identify potential participants. We will use multiple recruitment modalities including, but not limited to, recruitment through providers in Podiatry clinics, individual letters, posters in key clinics, electronic bulletin boards and My HealtheVet. We will track how participants heard about the study at enrollment in order to focus on the most successful recruitment modalities.

6.1.1 Distribution of IRB approved informational flyer

Study staff will distribute an IRB approved informational flyer/letter about the study to eligible potential participants and provide a phone number for follow-up. Study staff will approach eligible participants about participation using the following script.

Hello, my name is INSERT NAME HERE. I am a researcher with the Baltimore VA MC. I am part of a research team conducting a study on how to prevent foot ulcers in people with diabetes. You may be eligible to participate in the study. Would you like to hear more about it?

If NO, say the following:
Thank you for your time.

If YES, say the following:
The study is about how to prevent foot ulcers in people with diabetes through using a special wipe to clean your feet each day. You would have seven study visits over a little more than 1 year in which we will gently brush your feet with a swab like a Q-tip. This study will tell us whether using study cloth wipes prevents foot ulcers.

Are you interested in hearing more about the study?

If NO, say the following:
OK. Thank you for your time.

If YES:
Take the participant to a private space where no one can easily hear the conversation. Assess eligibility criteria and if eligible begin the informed consent process. If the participant is eligible and interested, but wants to take the consent home to review, make plans to follow-up with the potential participant.

Study staff will distribute an IRB approved informational flyer/letter about the study to VA providers. This flyer can be used to remind providers who may be eligible and how to refer potentially interested patients to us.

6.1.2 Mailing of an Introductory letter and an opt out card

Study staff will mail introductory letters to eligible participants to alert them about their potential eligibility for the study. They will also include an opt out card for potential participants

to return if they are not interested and do not wish to be contacted about the study. If study personnel do not receive the opt-out card from potential eligible participants, study personnel may contact the potentially eligible participants by phone.

6.2 Informed Consent

If the participant is willing to hear more about the project, study staff will verbally review the information on the IRB approved informational flyer. If the participant is interested, the study staff will schedule an in-person consent or a remote consent date. For in-person consent, the combined written informed consent/ HIPAA form will be discussed with the potential participant in a private area. For both in-person consent and remote consenting the potential participant will be asked if he/she wishes for study staff to read the consent form verbatim or to summarize it as he/she follows along. Potential participants will be asked a series of questions about the study to assess whether they understand what is involved in being in the study. Participants must answer all questions correctly in order to consent. Participants being consented remotely will be provided with 2 consent forms. The participant must sign and date both. They must either scan or take a picture of the signed consent page and transmit it to us using a VA approved Secure messaging program or they must mail one of the consents to us via the US postal service. The current approved messaging programs for transmitting documents with PHI/PII securely are Azure RMS or My HealtheVet Secure Messaging program. For participants being consented remotely, the enrollment visit may be split into 2. During the first part, the participant will be consented. The second part, remaining research procedures, will occur soon after the signed consent is received by the research team.

7 Study Intervention

Study participants will be assigned to our two experimental groups (chlorhexidine and placebo) using a blocked randomization with a 1:1 allocation ratio and variable block size at the time the participant returns for Visit #2 and agrees to continue participating in the study. The blocking will help assure that our study is balanced over time on the number of chlorhexidine and placebo participants. Participants randomized to the intervention will wash their feet using SAGE 2% CHLORHEXIDINE GLUCONATE CLOTHS to wipe down their feet each day and then apply supplied chlorhexidine-compatible over-the-counter moisturizer. The use of moisturizer is part of standard foot care for diabetes. Participants randomized to the control group will do the same except they will use SAGE COMFORT BATH CLOTHS which are the same cloths but do not contain chlorhexidine. The cloths will be matched in packaging, feel and smell to ensure a double blind.

The intervention will be stopped if the participant is felt to have a safety concern (e.g. allergy to study product). Because neither chlorhexidine nor mild soap will worsen wounds with inadvertent contact, the intervention can be used on existing wounds and ulcers. While unlikely, it is conceivable that a participant will need to be unblinded during the study. See the SOP for Unblinding a Participant for additional information.

7.1 Study Product Description

7.1.1 Acquisition

SAGE 2% CHLORHEXIDINE GLUCONATE CLOTHS, SAGE COMFORT BATH CLOTHS and a chlorhexidine-compatible over-the-counter moisturizer will be shipped to the research offices of the principal investigator.

7.1.2 Formulation, Packaging, and Labeling

SAGE 2% CHLORHEXIDINE GLUCONATE CLOTHS is an FDA-approved medication packaged and labeled by SAGE Products Inc. SAGE 2% CHLORHEXIDINE GLUCONATE CLOTHS is packaged with two 7.5in x 7.5in cloths to a package (3 sets of 2 cloth packages come bundled together). The product will be repackaged and labeled in a blinded fashion with seven cloths per package by study staff using a process that has been approved by the Baltimore VA investigational pharmacy.

SAGE COMFORT BATH CLOTHS contains eight 7.5in x 7.5in cloths soaked in mild, rinse-free cleansers and skin-protecting moisturizers like aloe and vitamin E per package. The product will be repackaged and labeled in a blinded fashion with seven cloths per package by study staff using a process that has been approved by the Baltimore VA investigational pharmacy.

The chlorhexidine-compatible over-the-counter moisturizer will be in its original packaging.

7.1.3 Product Storage and Stability

SAGE 2% CHLORHEXIDINE GLUCONATE CLOTHS and SAGE COMFORT BATH CLOTHS will be stored between 20-25°C (68-77°F), as directed by the manufacturers. The chlorhexidine-compatible over-the-counter moisturizer will be stored with the cloths. The study products (cloths and moisturizer) will be stored in a locked room in either a research office of the principal investigator or the Baltimore VA GRECC in a manner approved by the Baltimore VA investigational pharmacy.

7.2 Dosage, Preparation and Administration of Study Intervention

The cloths and moisturizer will be applied by the participant or caregiver once daily.

7.3 Modification of Study Intervention

If a participant develops signs of intolerance to either cloth or chlorhexidine-compatible over-the-counter moisturizer, they will be stopped, and the reaction evaluated. Signs of intolerance include generalized redness, persistent itching, or swelling of the feet in the absence of other identifiable causes. The VAMHCS will provide treatment as appropriate and the participant will return for evaluation. If clinically appropriate and the participant is willing, we will start a lower dose (e.g., wipe every other day) and reevaluate. If problem recurs, discontinue use of wipes. If problem does not recur increase to full dose. If problem recurs on full dose, repeat and keep on reduced dose.

7.4 Accountability Procedures for the Study Intervention

The PI is responsible for ensuring that a current record of product disposition is maintained, and product is dispensed only at an official study site by authorized personnel as required by applicable regulations and guidelines.

SAGE 2% CHLORHEXIDINE GLUCONATE CLOTHS/ SAGE COMFORT BATH CLOTHS and chlorhexidine-compatible over-the-counter moisturizer will be distributed to participants by trained study staff members. Documentation of all products received, distributed and destroyed will be kept by study staff members.

7.5 Assessment of Participant Compliance with Study Intervention

Instructions regarding dosing, missed doses and possible side effects will be provided. Adherence with cloths will be measured by 1) using chlorhexidine measurement of the skin from

non-invasive swabs of foot skin during each study visit, 2) counting remaining wipes in returned cloth wrappers and 3) having participants check off use on a monthly calendar. All will be returned to the study team during the in-person study visits. Participants doing remote study visits will report their number of remaining wipes over the phone, return their completed monthly calendars , and one additional bag of wipes will be prepared for each shipment to test confirmation of cloth type (chlorhexidine vs. placebo)

8 Study Procedures/Evaluations

	Visit # (approx. weeks)						
	1 (0)	2 (1)	3 (13)	4 (26)	5 (39)	6 (52)	7 (56)
Screen participants and verify inclusion and exclusion criteria that can be obtained at that time	X						
Review study with participant; administer questions to assure participant understands the study and their participation within the study.	X						
Obtain/Confirm informed consent	X						
Provide information on foot self-care for people with diabetes	X						
Confirm all inclusion and exclusion criteria		X					
Randomization		X					
Demonstrate use of cloths and moisturizer and have participant or family member demonstrate ability to use cloths; monitor for immediate allergic reaction		X					
Document current foot conditions, past foot conditions, usual foot self-care, type of footwear, recent antimicrobial use, complications of diabetes, control of diabetes and major medical issues including functional status from medical record and participant interview		X	X	X	X	X	
Record time and date of last time feet were washed. Record daily foot washing, drying, and foot lotion patterns from the last 4 weeks.	X	X	X	X	X	X	
Collect a focused history focusing on changes since enrollment		X	X	X	X	X	
Complete a brief physical assessment of the culture sites	X	X	X	X	X	X	
Obtain non-invasive skin specimens of nares and on each foot (Nares will not be collected if participant has respiratory symptoms)	X	X	X	X	X	X	X
Obtain photographs of each foot	X	X	X	X	X	X	X
Distribute SAGE 2% CHLORHEXIDINE GLUCONATE CLOTHS or SAGE COMFORT BATH CLOTHS and chlorhexidine over-the-counter compatible moisturizer daily x12 months		X	X	X	X	X	
Reconfirm participants willingness to participate		X	X	X	X	X	
Assess chlorhexidine levels on the feet using a non-invasive swab	X	X	X	X	X	X	X

	Visit # (approx. weeks)						
	**1 (0)**	**2 (1)**	**3 (13)**	**4 (26)**	**5 (39)**	**6 (52)**	**7 (56)**
Assess adherence with cloths participant diary, and counting all cloth wrappers (empty, partially filled or unused)							
Query participants for adverse events							
Physical assessment of the feet. Document development of a new foot ulcer or foot infection or other foot conditions. Document healing of existing foot wounds.							
Encourage adherence in use of study medication with up to weekly texts/phone calls		Up to weekly					
Call/text participant to inquire if any new concerns regarding foot health have developed		Monthly					
Medical record review for foot ulcers, infection or amputation; record clinical management of ulcers, infection or amputation	At week 52; participants with ulcers will be followed for additional 90 days if needed						

8.1 Study Visits

Study visit windows are as follows:

- Study visit #2 will occur 1 week after enrollment with a window of 3 to 28 days.
- Study visit #3 will occur 12 weeks after study visit #2 with a window of 5 to 18 weeks.
- Study visit #4 will occur 25 weeks after study visit #2 with a window of 19 to 31 weeks.
- Study visit #5 will occur 38 weeks after study visit #2 with a window of 32 to 44 weeks.
- Study visit #6 will occur 51 weeks after study visit #2 with a window of 45 to 53 weeks.
- Study visit #7 will occur at least 2 weeks after visit #6 or after they stop using study wipes. For participants who were randomized and self-withdraw or are randomized and are withdrawn from the study by study staff, visit #7 can occur at least 2 weeks after they stop using study wipes.

The participant interview portion of the study visit will be conducted by phone if participants are not able to make it into the VA during their scheduled visit. Participants that wish to do visits remotely will be asked to swab their feet and mail it to the lab. They will also be asked to take pictures of their feet and transmit it to study staff to assess foot health.

Missed visits will not be reported as deviations.

Participants will either be texted or called weekly, depending on the participant's preference as a reminder to use study medication. Participants who prefer to receive weekly reminder texts will have the option to receive them from the Annie App. The Annie App is a new VA approved texting program that provides a full audit trail of all text communications with participating veterans. We will also call participants monthly to assess foot health. Participants will also be encouraged to call if they have foot related issues. In the event there is a foot related issue, we will facilitate a visit to podiatry at the VAMHCS.

8.2 Laboratory Evaluations

During each study visit a specimen is obtained by trained research personnel from each of the following sites: anterior nares, and up to eight more swab areas on the feet. If a participant has an existing full thickness area of skin breakdown on one of the foot skin sites, research personnel will not obtain a specimen from that site. These specimens are tested for *S. aureus* and microbial burden using microbiological and PCR based methods and are also analyzed for the presence of all bacteria using metagenomics techniques. The swabs from visit # 2, #3, and #7 will be cultured for specific pathogens (*E. spp., Staphylococcus aureus, Klebsiella pneumoniae, Acinetobacter baumannii*, *Pseudomonas aeruginosa, and Enterococcus faecium* and diabetic foot infection pathogens) as well as *E. coli, Streptococcus, and Enterococcus faecalis*. Minimum inhibitory concentrations to chlorhexidine and to key antibiotics.

8.3 Clinical Evaluations

There will be a physical assessment of the feet at each study visit as well as photographs taken of the feet. Medical records will be reviewed to document the presence of new foot complications.

9 Unanticipated Problems and Serious Adverse Events

We will follow the reporting requirements specified in the VHA Handbook 1058.01. We will collect all of the information required to follow those reporting requirements. Reportable New Information[2] will be reported to the IRB within 5 business days.

10 Quality Control and Quality Assurance

The PI and affiliated institution are responsible for conducting the study in compliance with the IRB-approved protocol, all applicable federal and state regulations, VHA Handbook 1200.05 Requirements for the Protection of Human Subjects in Research and the International Conference on Harmonization, E6 Good Clinical Practice by adhering to the requirements for collecting, documenting and reporting complete and accurate data. A separate Data Quality Assurance Plan will be developed for this protocol to describe the frequency of review of study related data and the roles and responsibilities for activities related to Quality Control.

11 Statistical Plan

11.1 Analysis Plan

Primary objective: We hypothesize that the chlorhexidine group will have longer times to new foot complication event (chronic foot ulcer, foot infection, or foot amputation) than the placebo group. We will non-parametrically describe the distribution of "survival" times, from randomization until foot complication, in each group with a Kaplan-Meier curve as well as calculate the 75th percentile and median survival time, if possible. The log-rank test will test the null hypothesis of no difference in the distribution of times to foot complication between the two arms. In primary analyses, death will generally be treated as a non-informative censoring mechanism. Secondary analyses using more sophisticated models (e.g. Cox regression, partial competing risks model) will be utilized if there are significant prognosticator imbalances, death rates, or interval censoring Adherence with treatment will be examined as a potential moderator using Cox regression. A participant's medical chart will continue to be monitored for trial outcomes even if the patient stops participating in treatment (chlorhexidine or placebo) unless

[2] http://www.umaryland.edu/media/umb/oaa/hrp/documents/study-tools-docs/Reportable-New-Information_6-28-11.pdf

the patient withdraws consent for the study to continue to review their medical chart. Hence, generally the primary analysis will be intent-to-treat. For planning a larger clinical trial, we will also estimate the hazard ratio of new foot complications for chlorhexidine versus placebo with a 95% confidence interval.

Secondary objective: We hypothesize that a) the chlorhexidine group will not be colonized with *Enterobacter* spp., *S. aureus, K. pneumoniae, A. baumannii, P. aeruginosa* and *E. faecium* (ESKAPE) and diabetic foot infection pathogens with a higher minimum inhibitory concentration (MIC) to chlorhexidine than the placebo group. All study participants colonized with pathogens at the V7 time point will be included in the primary analysis. The null hypothesis of no difference in MIC distribution will be tested with the nonparametric Wilcoxon Rank Sum test due to the range-limited, discrete distributions of MICs. Effect sizes will be expressed in terms of means on a log(2) scale because the mean as an effect size metric will be more sensitive to group differences than the median. In addition, we will compute 95% confidence intervals around effect size estimates to quantify the precision of estimates.

Exploratory objective: This objective is not hypothesis driven. We will explore the impact of chlorhexidine on the diversity of the skin microbiota of the feet and the impact of the diversity of the skin microbiota on the development of new foot complications.

11.1 Sample Size Calculation

How many participants (or specimens, or charts) will be used in this study?
Local: 200 Worldwide: 200

Sample size is based on the primary objective and outcome. We will test the null hypothesis for the primary objective that there is no difference in time to new foot complication between the control and intervention groups using a log-rank test (two-tailed, 5% significance level). We assume that 10% of participants will die prior to one year of follow-up. We used the Freedman method for sample size calculation reported in Machin et al. (1997) using NCSS PASS software (2011). Prior data suggest that the annual proportion of individuals with new foot complications among controls will be about 0.4. If the true proportion for experimental participants is 0.2 one year after randomization, power of the log-rank test will be 0.82 to detect the difference if 100 patients are randomize to each study arm (i.e. total N=200).

12 Ethical Considerations

This study will be conducted according to US and international standards of Good Clinical Practice (FDA regulations 21 CFR 312 for IND studies and FDA guidance E6) for all studies. Applicable government regulations, VHA Handbook 1200.05 Requirements for the Protection of Human Subjects in Research and UMB research policies and procedures will also be followed.

All participants for this study will be provided a consent form describing this study and providing sufficient information for participants to make an informed decision about their participation in this study. This consent form will be submitted with the protocol for review and approval by the IRB. The formal consent of a participant, using the IRB-approved consent form, will be obtained before that participant is submitted to any study procedure. This consent form must be signed by the participant, and the investigator-designated research professional obtaining the consent.

13 Data Collection, Handling and Storage

The Principal Investigator is responsible to ensure the accuracy, completeness, legibility, and timeliness of the data reported. Data collection is the responsibility of the study staff. All

study personnel will be current in Information Security Training (VHA Privacy and Information Security and Rules of Behavior Training).

Paper records or case report forms will be filled out at the participating clinical sites or in the research team offices. Copies of the paper records or case report forms will serve as source documents and maintained for recording data for each subject enrolled in the study. All source documents will be completed in a legible manner to ensure accurate interpretation of data. When making changes or corrections, the original entry will be crossed out with a single line, and the change initialed and dated. Erasing, overwriting, or use of correction fluid or tape will not be done.

All source documents and laboratory reports will be reviewed by the clinical team and data entry staff, who will ensure that they are accurate and complete. AEs must be graded, assessed for severity and causality, and reviewed by the site PI or designee.

Confidentiality will be maintained to the extent permitted by law. Research records generated in this study will be transported from clinical protocol study site to data entry site by study personnel, accompanied by an Authorization to Transport and Utilize VA Sensitive Information Outside Protected Environments. Data will be maintained on a secure electronic central database on a VA research server (OITBALFPCRSCH01). Identifiable electronic study data will never be removed from behind the VA firewall. Analysis of identifiable study data will be performed only behind the VA firewall. Data will be backed up according to the VA network back up schedule. Paper records will be stored in locked filing cabinets behind a locked door in office space at the University of Maryland Baltimore that is accessible to the study PI. Identifiable records at UMB will only be accessible by VAMHCS staff on VA time. Study personnel who leave the research team will have their access to study data removed immediately. If data is lost/stolen, the VAMHCS, ISO/PO/PI and the UMB IRB will be notified immediately. A unique identifier or code will be assigned to each participant. Bacterial isolates and culture specimens and datasets used for analysis will be labeled with this code instead of the subject name or SSN. There will be no identifiable data on any of the samples or data sent to the UMB microbiology lab. Only the study personnel who directly interact with subject or manage the subject's clinical protocol data will have access to participant identifying information, which will be kept behind the VA firewall on the OITBALFPCRSCH01.VA05.med.va.gov server, in the folder \\\roghmann_share\Diabetic Foot Clinical Trial_HP79894\Databases\Main Study Database. Culture data will be entered directly into the relational database; however, microbiology laboratory personnel will not have access to participant identifiers in the database. Microbiological samples will be transferred to Dr. Roghmann's VA lab (Baltimore VA Medical Center, Microbiology Research Lab, 3C-112) on an ongoing, regular basis (approximately quarterly). The Principal Investigator will maintain all records pertaining to this study according to Records Control Schedule 10-1. As soon as permitted and when data collection is complete - source documents will be shredded and identifying information (names/SSN) will be removed from the database. There will be a single data table which maintains the link between the unique code and patient identifiers. This table will be maintained on a VA server. As soon as permitted and when data collection is complete, the link between code and identifying information will be deleted on the server and in any backups. If any paper copies of the link have been made, they will be shredded.

14 VA Informatics and Computing Infrastructure (VINCI)

The VA Informatics and Computing Infrastructure (VINCI) is a major informatics initiative of the Department of Veterans Affairs (VA) that provides a secure, central analytic platform for

performing research and supporting clinical operations activities. It is a partnership between the VA Office of Information Technology (OI&T) and the Veterans Health Administration Office of Research and Development (VHA ORD). VINCI includes a cluster of servers for securely hosting suites of databases integrated from select national VA data sources. VINCI servers for data, applications and virtual sessions are physically located at the VA Austin Information Technology Center (AITC), located in Austin, Texas. This secure enclave with 105 high-performance servers and 1.5 petabytes of high-speed data storage has multiple layers of security and disaster recovery to prevent data loss.

To ensure the protection of Veteran data, VINCI maintains compliance with the guidelines set forth by Veterans Health Administration (VHA) Handbook 1200.12, Use of Data and Data Repositories in VHA Research, and all other applicable VA and VHA policies and regulations. In addition, VINCI has undergone all security certification activities in support of obtaining an Authorization to Operate (ATO). Access to VINCI resources are approved in accordance with the requirements of National Data Systems (NDS), VHA Handbook 1200.12, Use of Data and Data Repositories in VHA Research, and all other applicable VA and VHA policies and regulations. All data transferred from VINCI is subject to audit for compliance.

VA-credentialed research or operations staff are granted access to study-specific data along with tools for analysis and reporting in the secure, virtual working environment through a certified VHA network computer within the VA. If not working within a VA or VHA hosted office environment containing VA network access, researchers may apply for and then access VINCI through an approved Virtual Private Network (VPN) and Remote Desktop application. The remote computing environment enables data analysis to be performed directly on VINCI servers, offering a number of advantages: uniform security standards for access; a common point of entry for all investigators who use the data; tools for analysis and reporting; tighter and more consistent control of data quality; and the ability to standardize and update terminology and format as technology and methodology improve.

Data Collection

VA provides care to Veterans at over 1,400 points of care. At the core of virtually all care processes is a broadly scoped and extensively used electronic health record system known as the Veterans Information System Technology Architecture (VistA). VistA provides a longitudinal view for patients receiving care nationwide including diagnoses, procedures, medications, labs, physiologic measurements, and text notes and reports. VA uses 130 VistA implementations to provide electronic health record services nationwide for just over 20 million Veterans historically. The aggregate content of these 130 VistA systems includes 2.3 billion documents (e.g., Progress Notes, Discharge Summaries, Reports) accumulating at a rate of 696,000 each day; 6.2 billion lab values (+1.5 million each day), 3.4 billion orders (+845,000 each day), and 1.7 billion medication administrations and prescription fills (+390,000 each day).

Data are aggregated from individual VistA systems to the VA Corporate Data Warehouse where it is modeled and prepared for use. Data published by the VHA Decision Support System (DSS), Inpatient and Outpatient Medical SAS (MedSAS), VA Health Economics Resource Center (HERC) cost data, Vital Status and VA-CMS linked data files maintained by VA Information Resource Center (VIReC), CDC National Death Index VA-linked data, and several other specialty data sets can be requested through VINCI. VA National Data Services and other data stewards regulate the right to use the data, but VINCI facilitates the process. When study

requests are approved, project-specific data are extracted from source databases and placed in SQL tables accessible only to the research team and VINCI data managers.

Storage of Study Data
Study data will be kept in accordance with the Department of Veterans Affairs Record Control Schedule 10-1 (RCS 10-1). Storage and transfer of any Personally Identifiable Information (PII) or Protected Health Information (PHI) must be done in accordance with applicable VA and VHA policies and directives, state and federal regulations, and applicable statutes including the Health Insurance Portability and Accountability ACT (HIPAA). Unless explicitly requested and approved by data stewards, all sensitive patient data must remain on VINCI project servers and only aggregate data without PII / PHI may be transferred from VINCI. Any desired change in data storage location or transfer requires amending the original data request with an updated of disposition of study data. The amendment must be approved by all data stewards before data may be transferred.

Violations of data policy or approved use of data will be subject to full penalty of law, which may include suspension of access privileges, reprimand, suspension from work, demotion, removal, and criminal and civil penalties.

Upon completion of the research project, the study principal investigator in conjunction with the VA Information Security Officer (ISO), and in accordance with VA policy, will ensure that, study data containing sensitive, confidential information will be returned to the VA, sanitized and removed from all servers, desktops, removable storage devices, etc.

Data Access
Only study team personnel explicitly authorized by data stewards will have access to project data. The study principal investigator has the responsibility for security of study. VINCI data managers and VA OI&T personnel not under the purview of the study principal investigator control the servers, network, processors, firewall and software in the VINCI environment, including access rights granted to study personnel.

When study personnel are no longer part of the research team, the study principal investigator will amend the data access request to terminate that person's access to all study data and notify the VA Information Security Officer of such action. No sensitive patient data may be shared with anyone who does not have a VA appointment. All study team personnel with access to sensitive patient data must stay current on required VA information security and privacy policy trainings.

Data Storage Location
Study data stored on VINCI servers is located at the Austin Information Technology Center, 1615 Woodward St., Austin, TX 78772-0001. The specific server where the data are stored within the VINCI environment will be chosen by VINCI personnel. The server name and location within the Austin Information Technology Center may be changed at any time at the discretion of VINCI personnel.

Specialized Software
All software used to access sensitive patient data, whether provided by VINCI, or developed by the study team, will run in virtual desktop sessions on VINCI servers within the Austin Information Technology Center.

15 Chesapeake Regional Information System for our Patients (CRISP)

CRISP is a regional health information exchange serving Maryland and the District of Columbia. The CRISP Portal is a free tool available to clinical staff. As clinical information is created and shared with CRISP, it is made accessible in real time to participating health providers through the CRISP Portal. The portal gives providers the ability to securely look up patient information though the internet. CRISP allows physicians and other authorized people to see healthcare activity at other hospitals. For this study, if an enrolled veteran seeks healthcare outside of the VA, researchers will be able to review the records in CRISP for that care to determine the study outcome. Identifying information for study participants will be securely transmitted between study personnel and CRISP using FIPS 140-2 validated encryption during transmission.

APPENDIX: Study variables

Variables	How Ascertained
**PARTICIPANT CHARACTERISTICS (measured once on each subject at Enrollment)**	
Name	CPRS
SSN	CPRS
Street address	Participant response
Phone number	Participant response
Email address and/or cell phone	Participant response
**Demographics**	
Age	CPRS
Sex	CPRS
Race	CPRS
Ethnicity	CPRS
Most recent height and date measured	CPRS
Most recent weight and date measured	CPRS
Able to use wipes for foot care	Participant response
Plan to reside in the area for the next 13 months	Participant response
Ability to walk	Participant response
Requires equivalent of institutional care	CPRS
Smoking status	Participant response
People in participant's household	Participant response
**Health Conditions**	
Diagnosis of diabetes	CPRS
Diabetes medication	CPRS
Year diabetes diagnosed	Participant response
Type of footwear used	Participant response
Presence of left foot	CPRS
Presence of right foot	CPRS
Current infection on either foot	CPRS/Foot photographs
Current foot ulcer, wound or unhealed amputation on left foot, date of diagnosis, size, location on foot	CPRS/Foot photographs
Current foot ulcer, wound or unhealed amputation on right foot, date of diagnosis, size, location on foot	CPRS/Foot photographs
Dates of healing of healed foot ulcers, wounds, infections and toe or transmetatarsal amputations (most recent captured for each)	CPRS
Amputation of the foot planned to treat current foot ulcer or wound	CPRS
Current preulcer lesion on either foot	CPRS/Foot photographs
Use of topical chlorhexidine or other topical antimicrobial agent on feet in past 7 days and in the past 12 months	Participant response
Use of systemic antibiotic in the past 3 months	CPRS
History of an allergic reaction to chlorhexidine	CPRS
Life expectancy >12 months	CPRS
Foot deformity/surgery	CPRS/Foot photographs

Variables	How Ascertained
Diabetes related co-morbidities such as PVD, congestive heart failure, etc	CPRS
Foot characteristics such as Charcot deformity and onychomycosis, etc	CPRS/Foot photographs
Current medications	CPRS
Visual acuity and retinopathy from most recent eye exam	CRPS
**Lab Values at Enrollment**	
Most recent HbA1c, date collected (also collected at subsequent study visits)	CPRS
Most recent LDL, date collected	CPRS
Most recent HDL, date collected	CPRS
Most recent creatinine, date collected	CPRS
Most recent total cholesterol, date collected	CPRS
**VARIABLES COLLECTED AT EACH VISIT**	
Currently smoke	Participant response
Frequency of foot washing with soap and water in past 4 weeks	Participant response
Frequency of foot washing with a washcloth in past 4 weeks	Participant response
Frequency of drying between toes in past 4 weeks	Participant response
Frequency of using lotion on feet in past 4 weeks	Participant response
Date and time of last foot washing	Participant response
Use of antimicrobials (other than study medication) since last study visit, dates of use	CPRS and participant response
Use of chlorhexidine wash on feet during podiatry visit	CPRS
Symptoms of allergic reaction to study medication	Participant response
Adherence to study medication	Participant adherence diary and count opened wipe packages
Development of a new foot ulcer	CPRS
Development of a new foot infection and if related to existing ulcer at randomization	CPRS
New foot amputation and if related to existing ulcer at randomization	CPRS
Healing of all foot ulcers, wounds, and unhealed toe or transmetatarsal amputations present at enrollment	CPRS
Nose and foot colonization results	Culture
Nose and foot microbiome results	Culture
Chlorhexidine level on foot skin	Culture
**VARIABLES COLLECTED AT EACH 'AT HOME' VISIT**	
Symptoms of allergic reaction to study medication	Participant response

Statistical Analysis Plan for the Primary and Secondary Endpoint
Preventing Diabetic Foot Ulcers through Cleaner Feet
VA DFU Clinical Trial – HP-79894
March 3, 2023

1. Aims

a. Primary. To determine if daily use of chlorhexidine on the feet for a year reduces new foot complications including chronic foot ulcer, foot infection or full or partial foot amputation.

b. Secondary. To determine if daily use of chlorhexidine on the feet for a year increases antibiotic resistance among bacterial pathogens on feet.

2. Endpoints

a. Primary. Time from randomization until either 1) a new chronic (present 28 days from initial diagnosis regardless of use of the intervention) foot ulcer or wound or 2) a moderate or severe foot infection (as defined by IDSA Diabetic Foot Infection Severity classification) not from an existing ulcer or 3) a full or partial foot amputation for a new ulcer. Participants are followed for the outcome from the date of randomization until either 53 weeks after randomization, the date of their V6 study visit, the date of their death or the date of their first outcome, whichever comes first.

b. Secondary. Minimum inhibitory concentration (MIC) to chlorhexidine and other key antibiotics of *E. cloacae, S. aureus, K. pneumoniae, A. baumannii, P. aeruginosa* and *E. faecium* (ESKAPE) and other diabetic foot infection pathogens.

3. Study Design

This is a single center, randomized, double blind (participant, outcome assessor), parallel group clinical trial comparing daily foot care with cloths containing 2% chlorhexidine compared to daily foot care with cloths not containing chlorhexidine. The target population is adults (age ≥ 18) with a clinical diagnosis of diabetes who are at risk (see protocol) for a new diabetic foot ulcer.

4. Randomization

Study participants will be assigned to two experimental groups (chlorhexidine and placebo) using blocked randomization with a 1:1 allocation ratio and variable block size. Randomization will not be stratified by site or other covariate.

5. Baseline Demographic and Clinical Characteristics

The study population description will include demographic characteristics, foot hygiene activities and relevant lab values at the time of enrollment and health indicators, comorbidities, recent medication use, current foot characteristics and past and current foot complications at the time of randomization. All descriptions will be stratified by treatment group. Participant characteristics will be described by mean and standard deviation, by median and interquartile range, or by frequency and percentage as appropriate.

6. Analysis Populations

a. Intention to Treat Population. All participants randomized into the study. Participants will be grouped based on the intervention that was assigned at randomization regardless of their participation in the intervention. Unless participants withdrew permission to access their medical records, outcome status based on medical record review

will be available on partially withdrawn participants. Data on fully withdrawn participants will be in the intention-to-treat analysis up to the time of withdrawal and then censored.

b. Per-Protocol Population. All randomized participants who were at least 80% adherent. Adherence is defined as the number of wipes the participant reported using divided by the number of days the participant was at risk of the outcome. Participants will be grouped based on the intervention that was assigned at randomization.

7. Handling of Missing Data

Due to low frequency of missing data, no imputation is planned.

8. Accounting for Multiple Comparisons

The test of the primary hypothesis is a single log-rank test with an alpha level of 0.05 (two-sided). Significance tests for the secondary aim will not be adjusted for multiple comparisons. The secondary outcomes (MICs to specific antimicrobials in specific bacterial pathogens) are safety outcomes and unadjusted p-values are more liberal in detecting a safety concern.

9. Analysis of the Primary Outcome

The primary analysis will be of the intent-to-treat population and will follow the principle of intent-to-treat. The primary hypothesis is specifically that the chlorhexidine group will have longer times to new foot complication event (chronic foot ulcer, foot infection, or foot amputation) than the placebo group and equivalently that the hazard rate for foot complications will be reduced in the chlorhexidine group. We will non-parametrically describe the distribution of event times, from randomization until diagnosis of foot complication, in each group with a Kaplan-Meier curve as well as calculate the 75th percentile and median event time, if possible. The log-rank test will test the null hypothesis of no difference in the distribution of times to foot complication between the two arms. In primary analyses, death will generally be treated as a non-informative censoring mechanism due to the low death rate over the observation period. Effect size will be measured with the hazard ratio and 95% confidence interval from a simple, unadjusted Cox regression model. The proportional hazards assumption will be assessed by analysis of residuals. A participant's medical chart will continue to be monitored for trial outcomes even if the patient stops participating in treatment (chlorhexidine or placebo) unless the patient withdraws consent for the study to continue to review their medical chart. For chronic foot ulcers, the event time will be time to first diagnosis of the ulcer that is still present 28 days later (i.e., that is ultimately chronic). Participants who develop a foot ulcer within the last 28 days of their time in the trial (observation period) will be followed to determine chronicity.

10. Secondary Analyses

a. Analysis of the Secondary Outcome

The null hypothesis that the chlorhexidine group will not be colonized with *E. cloacae, S. aureus, K. pneumoniae, A. baumannii, P. aeruginosa* and *E. faecium* (ESKAPE) and other diabetic foot infection pathogens with a higher minimum inhibitory concentration (MIC) to chlorhexidine than the placebo group will be tested with the nonparametric Wilcoxon Rank Sum test performed on normalized MICs from all detected ESKAPE organisms from each participant. MIC levels will be normalized by subtracting the MIC50 from the literature for each organism from the observed MIC on a $\log_2()$ scale. Effect sizes will be expressed in terms of means on a $\log_2()$ scale as the mean as an effect size metric will be more sensitive to group differences than the median. In addition, 95% confidence intervals will be calculated around effect size estimates to quantify the precision of estimates. All study participants colonized with pathogens at the V7 study visit will be included in the analysis. The V7 study visit occurred approximately 4 weeks after stopping the intervention. For participants who did not have the

primary outcome, the V7 study visit occurred approximately 13 months after randomization. For participants who had the primary outcome, the V7 study visit may have occurred sooner than 13 months after randomization.

b. Subgroup and Adjusted Analyses

The primary subgroup of interest is participants who had a resolved foot complication prior to randomization in the current study. The pre-specified subgroup will be defined specifically as participants with a prior foot complication defined as at least one of the following at any point in the past: foot ulcer or wound, partial foot amputation, or major foot infection. For the primary outcome (time until foot complication) a Cox regression model will be used to test whether there is a differential effect between randomized treatment groups between those who had and did not have a prior foot complication. Independent variables in this model will include treatment group indicator, subgroup indicator, and interaction between the group indicators. A significant interaction term would indicate a differential treatment effect depending on subgroup. Power will likely be subpar to detect an interaction, but hazard ratios and 95% confidence intervals will be presented overall and by subgroup as well. A similar strategy will be used for the secondary outcomes using linear regression, but again difference of means with 95% confidence intervals will be presented overall and by subgroup. Also, if there is a significant treatment arm imbalance on a variable predictive of the primary outcome, such as having a prior foot complication, the primary outcome will also be analyzed with Cox regression adjusting for this variable. An additional analysis will be to test the null hypothesis of no difference in healing between randomized treatment groups among participants with an existing foot ulcer at randomization. The outcome for this analysis is the healing of the existing foot ulcer prior to end of study follow up. A Fisher's exact test will be used to test this hypothesis.

13. Data Processing and Statistical Analysis Software

All final statistical analyses will be completed in Stata version 15 (College Station, TX), SAS (9.4) and R (4.2.1).

14. Sample Size Justification

Sample size is based on the primary objective and outcome. We will test the null hypothesis for the primary objective that there is no difference in time to new foot complication between the control and intervention groups using a log-rank test (two-tailed, 5% significance level). We assume that 10% of participants will die prior to one year of follow-up. We used the Freedman method for sample size calculation reported in Machin et al. (1997) using NCSS PASS software (2011). Prior data suggest that the annual proportion of individuals with new foot complications among controls will be about 0.4. If the true proportion for experimental participants is 0.2 one year after randomization, power of the log-rank test will be 0.87 to detect the difference if 100 patients are randomized to each study arm (i.e. total N=200).
