# Supplementary material for: Chlorhexidine vs Routine Foot Washing to Prevent Diabetic Foot Ulcers: A Randomized Clinical Trial
Source: JAMA Netw Open. 2025 Feb 18;8(2):e2460087. doi: 10.1001/jamanetworkopen.2024.60087 (PMC11836759; doi:10.1001/jamanetworkopen.2024.60087)
Supplement: Supplement 2. — eMethods. eTable. Chlorhexidine MIC50s From the Literature eReferences [file jamanetwopen-e2460087-s002.pdf]

Supplemental Online Content

Lydecker AD, Kim JJ, Robinson GL, et al. Chlorhexidine vs routine foot washing to prevent diabetic foot ulcers: a randomized clinical trial. *JAMA Netw Open*. 2025;8(2):e2460087. doi:10.1001/jamanetworkopen.2024.60087

eMethods.

eTable. Chlorhexidine MIC50s From the Literature

eReferences

This supplemental material has been provided by the authors to give readers additional information about their work.

© 2025 Lydecker AD et al. *JAMA Network Open*

eMethods.

Repackaging, Blinding and Testing of Study Wipes

Study wipes were repackaged in groups of 7 wipes into unmarked, opaque, resealable, heat-sealed bags for blinding participants and study staff using a standard operating procedure approved by the Baltimore VA Investigational Pharmacist. Each participant received a kit of 15 blinded bags of wipes at each study visit during the study period when the participant was supposed to be using wipes. Participants were assigned to sequentially numbered kits in order of randomization. This meant that the first randomized participant was assigned to kit number 1, the second randomized participant was assigned to kit number 2, etc. All kits looked identical with the exception of the kit number.

A sample of wipes from each kit was tested for the presence of chlorhexidine to ensure that each participant received the correct type of wipes throughout the study.

Chlorhexidine testing of wipes indicated that wipes from the correct treatment group were given to each participant for the duration of the study.

Instructions on How to Use Study Wipes and Lotion

Using the wipes:

Use one wipe once a day to clean all surfaces of your feet below the ankle, including the underside of both of your feet and in between each of your toes. If you have a wound or existing ulcer which requires a dressing, use the wipe on the exposed part of your feet each day. You can wipe the skin around the wound or ulcer when the dressing is changed. It is all right if the wipe touches the wound or ulcer.

If you miss using a wipe to clean your feet for a day or several days, do not double up on wipes to try to make up for missing days.

Using the lotion:

Wait for your feet to dry after using the wipe, then apply a dime-sized amount of lotion to each foot.

Adherence calendar:

© 2025 Lydecker AD et al. *JAMA Network Open*

Fill in the circles or mark the circles with checks on the days that you use the study wipes and lotion. Please be accurate with days you are using the study wipes and lotion so that study results are reliable.

Returning wipes and empty packs:

Once you have used an entire pack of 7 wipes, save the empty pack to return to the study team at your next visit. During your next study visit, return any unused, partially used, and empty packs to the study team.

If you miss your next study visit, save all unused, partially used and empty packs until you communicate with the study team. The study team will be shipping you wipes as needed as long as you indicate you still wish to be part of the study.

Possible side effect:

Mild skin irritation or allergic reaction may occur, although this is rare. Contact the study team if you feel you are having a side effect.

Details on Swab Work Up for ESKAPE Pathogens

This was done by enriching 50 µl of the E-swab solution in Tryptic Soy Broth with 6.5% salt (TSB with salt) broth and in Brain Heart infusion (BHI) broth. Fifty µl of TSB with salt broth was then plated onto a blood agar plate and Phenyl-Ethyl Alcohol plate and 50µl of BHI broth was plated onto MacConkey plates. Unique colony types were isolated and worked up for the pathogens listed above. The minimum inhibitory concentration (MIC) to chlorhexidine for each of these isolates was ascertained using microbroth dilution.[1,2]

eReferences

1. Hayden MK, Lolans K, Haffenreffer K, et al. Chlorhexidine and Mupirocin Susceptibility of Methicillin-Resistant Staphylococcus aureus Isolates in the REDUCE-MRSA Trial. J Clin Microbiol. 2016;54(11):2735-2742.

2. Clinical and Laboratory Standards Institute. Methods for Dilution Antimicrobial Susceptibility Tests for Bacteria That Grow Aerobically; Approved Standard. CLSI Document M07. Eleventh Edition. Clinical and Laboratory Standards Institute; 2018.

© 2025 Lydecker AD et al. *JAMA Network Open*

Details on Swab Work Up for Presence of Chlorhexidine

These swabs were analyzed using a colorimetric assay[1,2] within 24 hours of collection.

Prespecified Subgroup Sensitivity Analysis

The pre-specified subgroup was participants with a prior resolved foot complication (foot ulcer or wound, partial foot amputation, or major foot infection) at any point in the past. To examine whether there was a differential treatment effect between those with and without prior resolved foot complication, we added the indicator variable for prior resolved foot complication and the interaction term between this indicator and treatment group. A significant interaction term would indicate that the treatment effect among those with a prior resolved foot complication differs from those without a prior resolved foot complication.

eTable. Chlorhexidine MIC50s From the Literature

Pathogen	Chlorhexidine MIC50	Reference
Staphylococcus aureus	2	Lutgring(3)
Acinetobacter baumannii	32	Kampf(4)
Pseudomonas aeruginosa	16	Kampf(4)
Enterococcus faecalis	32	Morrissey(5)
Enterococcus faecium	8	Morrissey (5)
Enterobacter cloacae complex (includes Enterobacter cloacae and Enterobacter asburiae)	16	Lutgring(3)
Escherichia coli	2	Lutgring(3)
Klebsiella pneumoniae	16	Lutgring(3)

eReferences

1. Supple L, Kumaraswami M, Kundrapu S, et al. Chlorhexidine Only Works If Applied Correctly: Use of a Simple Colorimetric Assay to Provide Monitoring and Feedback on Effectiveness of Chlorhexidine Application. Infect Control Hosp Epidemiol. 2015;36(9):1095-1097. doi:10.1017/ice.2015.124

2. Edmiston CE, Krepel CJ, Seabrook GR, Lewis BD, Brown KR, Towne JB. Preoperative shower revisited: can high topical antiseptic levels be achieved on the skin surface before

© 2025 Lydecker AD et al. *JAMA Network Open*

surgical admission? J Am Coll Surg. 2008;207(2):233-239. doi:10.1016/j.jamcollsurg.2007.12.054

3. Lutgring JD, Grass JE, Lonsway D, Yoo BB, Epson E, Crumpler M, et al. Development of a Broth Microdilution Method To Characterize Chlorhexidine MICs among Bacteria Collected from 2005 to 2019 at Three U.S. Sites. Microbiol Spectr. 2023 Jun 15;11(3):e0413422.

4. Kampf G. Acquired resistance to chlorhexidine - is it time to establish an “antiseptic stewardship” initiative? J Hosp Infect. 2016;94(3):213–27.

5. Morrissey I, Oggioni MR, Knight D, Curiao T, Coque T, Kalkanci A, et al. Evaluation of epidemiological cut-off values indicates that biocide resistant subpopulations are uncommon in natural isolates of clinically-relevant microorganisms. PLoS One. 2014;9(1):e86669.

© 2025 Lydecker AD et al. *JAMA Network Open*
