# Supplementary material for: Chlorhexidine vs Routine Foot Washing to Prevent Diabetic Foot Ulcers: A Randomized Clinical Trial
Source: JAMA Netw Open. 2025 Feb 18;8(2):e2460087. doi: 10.1001/jamanetworkopen.2024.60087 (PMC11836759; doi:10.1001/jamanetworkopen.2024.60087)
Supplement: Supplement 3. — Data Sharing Statement [file jamanetwopen-e2460087-s003.pdf]

Data Sharing Statement

Lydecker. Chlorhexidine vs Routine Foot Washing to Prevent Diabetic Foot Ulcers. *JAMA Netw Open*. Published February 18, 2025. doi:10.1001/jamanetworkopen.2024.60087

Data
Additional Information: Registered in ClinicatTrials.gov (NCT03503370): https://clinicaltrials.gov/study/NCT03503370
Data available: Yes
Data types: Deidentified participant data, Data dictionary
How to access data: Deidentified participant data and a corresponding data dictionary will be posted to a freely available repository (ex figshare) upon acceptance of this manuscript. We would like to post the data AFTER acceptance in case reviewers would like an additional variable added to the analysis (and therefore that variable would need to be added to the data and data dictionary that we post).
When available: With publication

Supporting Documents
Document types: None

Additional Information
Who can access the data: The data will be fully deidentified and freely available to anyone via the data repository.
Types of analyses: For any purpose.
Mechanisms of data availability: Data will be freely available
